# Path-Based Nonequilibrium Binding Free Energy Estimation, from Protein–Ligand to RNA-Ligand Binding

**DOI:** 10.1021/acs.jcim.5c00452

**Published:** 2025-06-06

**Authors:** Eleonora Serra, Alessia Ghidini, Riccardo Aguti, Mattia Bernetti, Sergio Decherchi, Andrea Cavalli

**Affiliations:** † Department of Pharmacy and Biotechnology (FaBiT), Alma Mater Studiorum, University of Bologna, via Belmeloro 6, 40126 Bologna, Italy; ‡ Computational & Chemical Biology, 121451 Fondazione Istituto Italiano di Tecnologia, via Morego 30, 16163 Genoa, Italy; § Centre Européen de Calcul Atomique et Moléculaire (CECAM), Ecole Polytechnique Fédérale de Lausanne, 1015 Lausanne, Switzerland; ∥ Department of Biomolecular Sciences, University of Urbino “Carlo Bo”, Piazza Rinascimento 6, 61029 Urbino, Italy; ⊥ Data Science and Computation Facility, Fondazione Istituto Italiano di Tecnologia, via Morego 30, 16163 Genoa, Italy

## Abstract

In this study, we addressed the challenge of estimating binding free energies in complex biological systems of pharmaceutical relevance, including both protein–ligand and RNA-ligand complexes. As case studies, we examined the intricate binding of the drug Gleevec to Abl-tyrosine kinase and two ligands binding to the preQ1 RNA riboswitch. By refining our approach based on nonequilibrium steered molecular dynamics simulations and path-based collective variables, we tackled the specific difficulties posed by these systems. In particular, the Abl–Gleevec complex is characterized by significant system size and extensive conformational rearrangements of the protein, whereas the systems involving RNA are characterized by marked conformational flexibility. For the Abl–Gleevec system, our method produced binding free energy estimates closely aligned with experimental values, demonstrating its reliability. For the RNA-ligand complexes investigated, we found that the simpler water model TIP3P yields more accurate free energy estimates than the TIP4P-D model, offering practical insight for future research. In this case, the agreement with the experimental results is reasonable. Overall, this work underscores the effectiveness of the proposed path-based workflow in handling complex biomolecular systems with unique characteristics, enabling systematic binding free energy predictions across a variety of targets.

## Graphical abstract

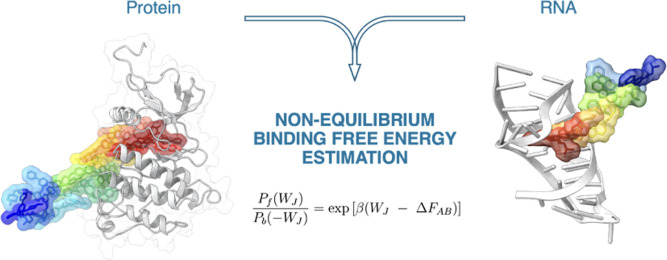

## Introduction

The binding free energy (Δ*F*_b_) quantifies the affinity of a potential drug for its biological target, making Δ*F*_b_ a central thermodynamic observable in drug discovery campaigns.^1^ During the past few decades, numerous computational methods have been developed to estimate Δ*F*_b_, yet accurately predicting this parameter remains a challenge in many cases. The difficulties stem from various aspects, including the high degrees of freedom of the systems and the inherent flexibility of both the receptor and the ligand.

Motivated by the goal of developing a systematic protocol for computing binding free energies with path-based methods, we recently presented a semiautomatic computational workflow using nonequilibrium simulations,^2^ successful on systems of moderate size. However, challenges emerged when dealing with intricate systems, such as the Abl–Gleevec complex, which induced a very high work dissipation, hence poor convergence of free energy estimates.^2^ To improve convergence in large systems, we developed a refined strategy aimed at mitigating dissipation.^3^ In this work, taking advantage of this improved nonequilibrium strategy for binding free energy estimation,^3^ we now deal with intricate protein and RNA targets. Specifically, we take the Abl-tyrosine kinase-Gleevec complex and two RNA-ligand complexes as test cases, showcasing the adaptability of our computational workflow. Additionally, for the RNA case, we propose an ad-hoc approach to generate accurate unbinding paths via electrostatics.

The 2-phenylaminopyrimidine derivative Imatinib, commercially known as Gleevec, is an anticancer marketed drug that structurally resembles the natural substrate ATP of Abl kinase, allowing it to bind to the ATP-binding site of the kinase. Upon binding, Imatinib inhibits this site, blocking the enzymatic activity of the kinase. From a computational drug design perspective, modeling the binding and unbinding of Imatinib to Abl kinase poses significant challenges. This is mainly due to the extensive protein rearrangements that distinguish the active state of Abl kinase, which is the predominant conformation in solution, from the inactive state it adopts upon binding to Imatinib^4^ (Figure 2). Key changes occur to the activation loop (A-loop) of Abl, which controls access to the active site and contains a tyrosine residue that is phosphorylated to modulate activity.^5^ In the Abl active state, the A-loop is in an open conformation that permits the binding of substrates (ATP or ADP) and their phosphorylation. In contrast, when Abl binds Imatinib, the A-loop experiences a rotation that results in the movement of up to 35 Å for central residues. Additionally, a distinctive DFG (Asp-Phe-Gly) motif within the kinase activation loop undergoes a 180° rotation in the inhibited structure with Imatinib compared to the uninhibited form. In this latter, this motif adopts the DFG-in conformation, where the DFG Asp residue points into the active site, allowing it to coordinate with Mg-ATP. However, in the structured inhibited by Imatinib, the DFG motif is in the out conformation, causing the Phe of the DFG motif to flip into the catalytic pocket.

The ability of Imatinib to induce substantial protein conformational changes, involving more than 20 residues, is closely related to its therapeutic impact. An additional complexity is due to the high flexibility and large size of the ligand Imatinib. Nonetheless, investigating the interactions and binding/unbinding mechanisms of the Abl–Gleevec complex is of high biological and pharmaceutical significance.^6^ To this end, our nonequilibrium protocol based on physical pathways may represent an ideal choice to compute the binding free energy of this complex, as it can provide insights into the underlying interactions and mechanisms. Consequently, the Abl–Gleevec complex is used in this work as representative case of a challenging and pharmaceutically relevant protein–ligand complex.

The challenges faced in the Abl–Gleevec system mirror the complexity encountered in the targeting of RNA molecules. The latter have recently gained increasing interest as promising pharmaceutical targets of small molecules,^7–12^ boosted by the recent FDA approval of Risdiplam,^13,14^ a small molecule drug targeting RNA. The peculiar features of RNA molecules, such as complex structural dynamics and high charge density, along with the limited knowledge about RNA-ligand interactions, make them challenging targets for both experimental and computational approaches.^15–17^ Thus, further investigations in RNA-ligand recognition are highly desirable and may promote tangible advancements toward the design of RNA-targeting small molecule drugs. In this context, RNA riboswitches^18^ are emerging as a compelling class of RNA targets,^19–21^ as they are able to bind metabolites and modulate gene expression as a result. In particular, since they are mainly found in bacteria, including human pathogens, they are gaining relevance in the urgency of bacterial resistance, which is recognized by the World Health Organization (WHO) as a major threat.^19,20^ A concrete example is given by the campaign against the bacterial FMN riboswitch, with a compound at the preclinical stage.^22^ Given the availability of experimental structures in complex with different ligands, along with experimental affinity data,^23,24^ we take the preQ1 riboswitch as a test case of our procedure in the context of RNA-ligand binding.

Overall this work demonstrates the applicability of the path-based method proposed to protein–ligand systems with realistic complexity, as well as the emerging class of RNA-ligand partners. This approach is designed to be systematically applicable, highlighting its potential for drug discovery endeavors.

## Methods

The main steps of our nonequilibrium strategy combining Steered Molecular Dynamics (SMD), Path Collective Variables (PCVs) and the Crooks Fluctuation Theorem (CFT) are summarized here and detailed in the following subsections:

1. Generation of unbinding MD trajectories starting from a bound complex through Adiabatic Bias MD (ABMD)^25^ coupled with an electrostatic-like collective variable (CV)^26^ to promote protein–ligand dissociation. Among several trajectories generated, the one representing the most probable and plausible mechanism is chosen as the guess path.
2. The guess path is further optimized using two path algorithms, namely the Principal Path Algorithm^27^ and the Equidistant Waypoints Algorithm,^2^ resulting in an optimized reference path to be used in the subsequent simulations.
3. Multiple replicates of bidirectional (i.e., binding and unbinding) nonequilibrium SMD simulations are conducted following the reference path via the PCVs. The Jarzynski work (*W*_J_) performed during the simulations is calculated.
4. The Free Energy Surface (FES) is computed by applying the CFT piece-wise along the path to the *W*_J_ values collected during binding and unbinding SMD simulations.^2^
5. Finally, the standard binding free energy is estimated as the sum of the binding free energy obtained by the ratio of the bound and unbound partition functions along the FES and a correction term due to the accessible ligand volume with respect to the standard volume.^28^

A schematic representation of the computational pipeline is reported in Figure 1.

**Figure 1:**
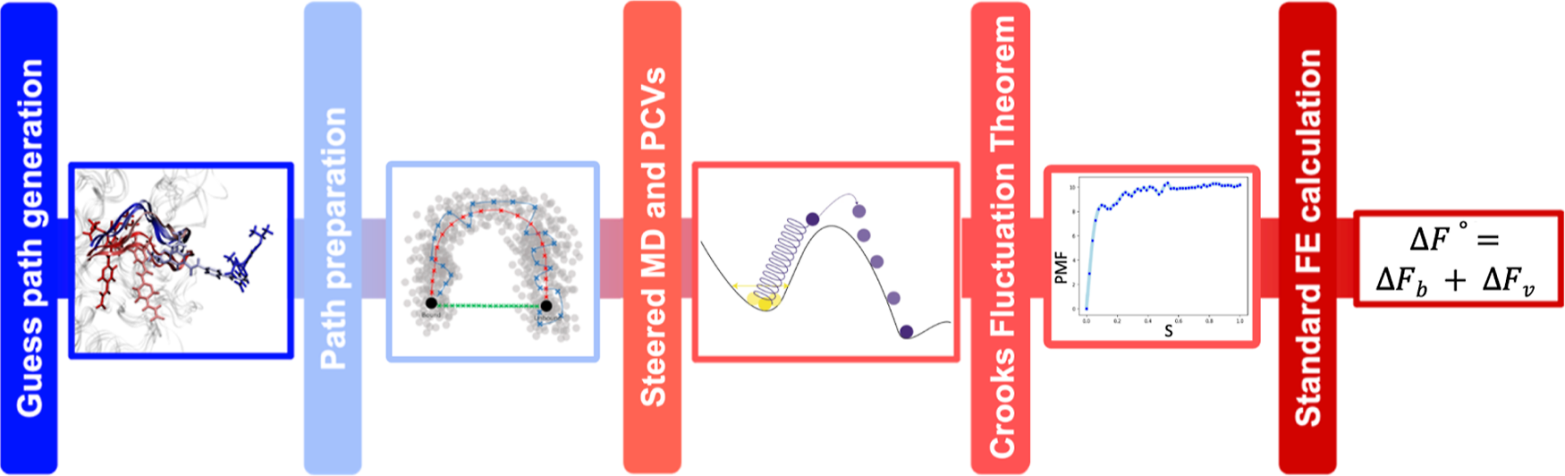
Schematic representation of the computational pipeline based on nonequilibrium SMD simulations and the Crooks Fluctuation Theorem.

The strategy relies on Steered Molecular Dynamics,^29^ where a time-dependent harmonic potential is added to the regular system potential. Several trivially parallel replicates of SMD simulations are performed to sample the unbinding/binding process following the predefined physical pathway connecting the bound and unbound states. As bidirectional simulations are performed, the Crooks Fluctuation Theorem^30^ can be used to determine the FES, leveraging data from both binding and unbinding simulations. The FES is the Landau free energy profile along the collective variable used to perform the enhanced sampling simulation, in this case *S*(*x*).

### Adiabatic Bias MD Simulations

The first step in the pipeline is the definition of PCVs.^31^ To identify PCVs for each studied system, multiple MD trajectories of ligand unbinding from the native bound pose, as found in the respective X-ray crystallographic complexes, were produced with the enhanced sampling method ABMD.^25^ In ABMD, a target value for the collective variable is defined and achieved thanks to a moving harmonic restraint that is active only when the collective variable is not progressing toward the defined value. The main advantage of using ABMD to derive guess trajectories instead of other enhanced sampling methods lies in its gentleness, as the restraint center progresses toward the target CV value due to thermal fluctuations only.^25^

For protein–ligand systems, starting from the bound complex, the unbinding process is sampled with ABMD using an electrostatic-like CV. Specifically, this CV consists in a fictitious electrostatic potential generated by fictitious charges of the same sign placed on the ligand and the protein. The target value of this fictitious potential is set to zero, corresponding to ligand dissociation. An ABMD force constant of 10^–17^ (kJ/mol)^−3^ was employed. Conversely, for the RNA-ligand complexes we took advantage of the inherent electrostatic of the systems. Given the highly negative nature of the RNA electrostatic potential and the positive charge in the ligands, ABMD simulations were run employing a CV based on the Debye–Hückel interaction energy. Through this strategy, we guided the systems toward a zero electrostatic-interaction energy between the RNA and the ligands, leading to complete dissociation of the two partners. Force constants of 10^–1^ and 10^–2^ (kJ/mol)^−3^ were used in the ABMD simulations.

For each complex, several ABMD simulations were carried out, aiming to capturing all possible unbinding pathways. Among these, the trajectory observed with the highest probability is chosen as the guess path. In the Results and Discussion section, we discuss additional criteria that we found relevant to select an optimal guess path.

The selected trajectory of each complex is optimized with two path algorithms: the principal path algorithm, which optimizes the path in the configurational space from an initial bound state to a final unbound state, and a refined version of the equidistant waypoint algorithm, which equispaced the mean-square-deviation (MSD) between subsequent configurations of the path, as required when using PCVs. As a result, a smooth path composed of consecutive equidistant configurations in terms of MSD (with an average MSD between subsequent frames of 1 Å^2^) connecting the bound and unbound states is obtained.

### Path Collective Variables (PCVs)

In order to use the CFT estimator, SMD simulations must be performed bidirectionally (i.e., simulating the binding and unbinding) using the same protocol. The PCVs developed by Branduardi et al.^31^ are ideal for this task, as they enable mapping the position of a point in the configurational space relative to a predefined path. Therefore, PCVs can be used to follow a predetermined pathway forward and backward, avoiding ambiguities in the evolution of the system. PCVs consist of *S*(*x*), which measures the progression along the predefined path, and *Z*(*x*), which measures the orthogonal deviation from the pathway

1 $$S\left(x\right)=\frac{\sum_{i=1}^{p}ie^{-\lambda {∥x-x_{i}∥}^{2}}}{\sum_{j=1}^{p}e^{-\lambda {∥x-x_{j}∥}^{2}}}$$

2 $$Z\left(x\right)=-\lambda^{-1}\;\ln\sum_{i=1}^{p}e^{-\lambda {∥x-x_{i}∥}^{2}}$$

where *p* is the number of molecular structures included in the reference path and ${∥x-x_{i}∥}^{2}$ measures the distance (herein the MSD in the Cartesian space) between the *i*th configuration in the path (*x*_*i*_) and the instantaneous microscopic configuration (*x*), while λ controls the smoothness. The *S*(*x*) PCV can be seen as an indicator function of the configuration progression, as when *x* ≈ *x*_*i*_ then *S*(*x*) will have approximately the integer value *i*. To achieve a correct mapping, λ can be parametrized as follows

3 $$\lambda =\frac{2.3p}{\sum_{i=1}^{p-1}{∥x_{i}-x_{i+1}∥}^{2}}$$

where ${∥x_{i}-x_{i+1}∥}^{2}$ measures the distance between the *i*th and the (*i* + 1)­th configuration and *p* is the number of configurations in the reference path.^32^ *S*(*x*) can assume values from 1 to *p*; however, in this study, *S*(*x*) is normalized to a range of 0 to 1.

### SMD Simulations

To sample the binding/unbinding events, SMD simulations with PCVs are performed. In SMD simulations, a time-dependent harmonic restraint *R*(*x*, *t*) is applied along the pulling coordinate *Ŝ*(*t*)­

4 $$R\left(x,t\right)\equiv \frac{1}{2}k{\left(S\left(x\right)-Ŝ\left(t\right)\right)}^{2}$$

Additionally, a half-harmonic wall (flat-bottom potential) is employed to confine the system along *Z*. This upper wall is active only when the *Z*(*x*) PCV value surpasses a threshold of *Z* = 0.05 nm^2^.

A key quantity to be introduced is the Jarzynski work (*W*_J_).^33^ This is the path integral of $\mathrm{\xi ̇}\partial H_{\xi }/\partial \xi$ along the trajectory Γ_*t*_

5 $$W_{J}=\int_{0}^{t_{s}}\;dt\mathrm{\xi ̇}\frac{\partial H_{\xi }}{\partial \xi }\left(\Gamma_{t}\right)$$

where *H*_ξ_ represents the time-dependent part of the Hamiltonian that is added to the regular potential in SMD and ξ is the time-varying variable. The Jarzynski work of a simulation in the canonical ensemble accounts for the work required to transition the system from the initial to the final state (or vice versa).^34^

According to the second law of thermodynamics, in a quasi-static transformation, this amount of work corresponds to the free energy difference between states *A* and *B* of the system, Δ*F*_AB_ = *F*(*B*) – *F*(*A*). However, for nonequilibrium irreversible transformations, the total Jarzynski work will, on average, exceed the free energy difference by a quantity known as the dissipated work (*W*_J_^diss^)^33^

6 $$W_{J}^{diss}=\left\langle W_{J}\right\rangle -\Delta F_{AB}\geq 0$$

During SMD simulations, the higher the pulling speed, the greater the total *W*_J_, and in turn the higher the dissipated work. Several binding and unbinding nonequilibrium simulations with a specific pulling speed are necessary for free energy estimation. The SMD restraint moves at constant velocity, thus the simulation length is inversely proportional to the pulling speed.

The pulling speed and the number of SMD replicas for each system were chosen to ensure convergence of free energy estimates. For all complexes, 30 binding and 30 unbinding simulations of 100 ns were required. Moreover, to ensure consistency across different pulling speeds, the SMD simulations of the Abl–Gleevec system were performed also with a time length of 200 ns. The force constant of the moving harmonic restraint applied during the SMD is 20 kJ/mol for both systems.

### FES Estimation

The free energy profile along *S*(*x*) is reconstructed from SMD simulations using the bidirectional nonequilibrium CFT estimator.^30,35^ According to the Crooks Fluctuation Theorem

7 $$\frac{P_{f}\left(W_{J}\right)}{P_{b}\left(-W_{J}\right)}=\exp\left[\beta \left(W_{J}-\Delta F_{AB}\right)\right]$$

where β = 1/*k*_B_ *T*, *k*_B_ is the Boltzmann constant, and *T* is the absolute temperature of the simulated system. *P*_*f*_(*W*_J_) and *P*_*b*_(−*W*_J_) are the forward *W*_J_ and backward −*W*_J_ distributions, respectively. Specifically, to compute the free energy profile we relied on a maximum likelihood interpretation of the CFT^36^ based on the Bennett algorithm.^2^ As we perform an equal number of forward and backward SMD replicates with PCVs along the same reference pathway, free energy differences can be estimated by solving self-consistently the following equation

8 $${\left\langle {\left\{1+\exp\left[\beta \left(W_{J}^{f}-\Delta F_{S_{i}S_{i+1}}\right)\right]\right\}}^{-1}\right\rangle }_{A\to B}={\left\langle {\left\{1+\exp\left[-\beta \left(W_{J}^{b}-\Delta F_{S_{i}S_{i+1}}\right)\right]\right\}}^{-1}\right\rangle }_{B\to A}$$

where the work values from forward and backward simulations, *W*_J_^f^ and *W*_J_^b^, are related to the *S*_*i*_, *S*_*i*+1_ interval. The FES point *F*(*S*_*i*+1_) corresponds to $F\left(S_{i}\right)+\Delta F_{S_{i}S_{i+1}}$ , where the *i* index identifies the *i*-th configuration of the reference path. Therefore, solving eq 8 for progressively increasing values of *i* results in the complete free energy profiles along *S*(*x*). As we normalized *S*(*x*) between 0 and 1, *S*(*x*) = 0 corresponds to the bound state, while *S*(*x*) = 1 to the unbound state.

Finally, to enable a comparison with the estimates obtained from the bidirectional method, unidirectional Jarzynski estimates can be calculated using a similar procedure and the Jarzynski equality^37^

9 $$\Delta F_{AB}=-\frac{1}{\beta }{\left\langle \exp\left(-\beta W_{J}^{f}\right)\right\rangle }_{f}$$

Here, the angular brackets denote an exponential average taken over *N* nonequilibrium trajectories, in which the system transitions from its initial state to the target state, following an identical protocol.

### Standard Binding Free Energy Estimation

To compare the results of the protocol with experimental values, we calculate the standard binding free energy (Δ*F*_b_°), namely the sum of the binding free energy (Δ*F*_b_) and the standard volume correction term (Δ*F*_v_), as described, for instance, by Doudou et al.^28^

10 $$\Delta {F_{b}}^{{}^{\circ}}=\Delta F_{b}+\Delta F_{v}=-\frac{1}{\beta }\;\ln\;\frac{Q_{site}}{Q_{bulk}}-\frac{1}{\beta }\;\ln\frac{V_{bulk}}{V^{{}^{\circ}}}$$

Here, Δ*F*_b_ is the ratio between the probabilities of the bound and unbound ligand states, i.e. of the canonical partition functions of the bound (*Q*_site_) and unbound (*Q*_bulk_) states. Specifically, Δ*F*_b_ is determined by integrating the FES along *S*(*x*) in the bound and unbound regions

11 $$\frac{Q_{site}}{Q_{bulk}}=\frac{\int_{site}\;\exp\left(-\frac{F\left(S\right)}{RT}\right)dS}{\int_{bulk}\;\exp\left(-\frac{F\left(S\right)}{RT}\right)dS}$$

This approach requires identifying the specific molecular configuration in the reference path that discriminates between the bound and unbound regions. The *S*(*x*) value corresponding to such molecular configuration is selected through analysis of the FES followed by visual inspection of trajectories, relying on the protocol we outlined in ref 2.

The second term in eq 11 is the standard volume correction term, Δ*F*_v_. It quantifies the variation of the free energy due to considering the standard-state volume *V*° corresponding to 1661 Å^3^ (concentration of 1 M) instead of the effectively sampled unbound volume *V*_bulk_. This contribution is computed using NanoShaper.^38^ In detail, we first isolated the set of ligand configurations of the reference pathway associated with the unbound state. On the union (namely, aggregation of the pdb files) of these configurations, we computed the solvent excluded surface. The resulting volume is a proxy of the unbound volume spanned by the ligand in the unbound state.

Finally, binding free energy errors are calculated via bootstrap analysis.^39^

### Well-Tempered Metadynamics Simulations

To further assess the reliability of our results, we repeated the calculations with an established protocol,^40–43^ namely Well-Tempered Metadynamics (MetaD) simulations^44,45^ coupled with PCVs.^31^ The deposition time of Gaussians was set to 250 MD steps, and a bias factor of 15 was selected. Gaussian height of 1.0 kcal/mol was used, while Gaussian width was set to 0.2 along *S*(*x*) and 0.01 nm^2^ along *Z*(*x*). As for SMD simulations, the orthogonal deviation for the reference path was restricted with a wall at *Z* = 0.05 nm^2^ with force constant of 4 ×10^7^ kJ mol^–1^ nm^–4^.

Production runs resulted in 1 μs simulations for the two Riboswitch-ligand systems, and 1.5 μs for the Abl–Gleevec complex. Convergence was determined based on two conditions: the full diffusivity of the system along the PCV *S*(*x*), and the residual Gaussian height being less than 10% of the initial height, aligning with previous methodologies.^46^

For each system, we derived the FES from the Well-Tempered MetaD simulations, and the free energy was set to zero at the lowest point, corresponding to the ligand bound state. To estimate the standard binding free energies from the FES, the same procedure used for the SMD simulations was applied. The statistical errors on the standard binding free energies estimated from the Well-Tempered MetaD were computed via bootstrap, after dividing the MetaD simulation in 10 blocks and using 400 bootstrapping iterations.

### Setup of the Systems

The Abl kinase is composed of a small N-terminal lobe, consisting of five β-strands and an α-helix, and a larger C-terminal lobe, made up of multiple helices. The ATP-binding site is positioned between these two lobes, with a cleft between them. The X-ray structure of the Abl–Gleevec complex is represented in Figure 2 and compared to an X-ray structure of Abl in an active conformation.

**Figure 2:**
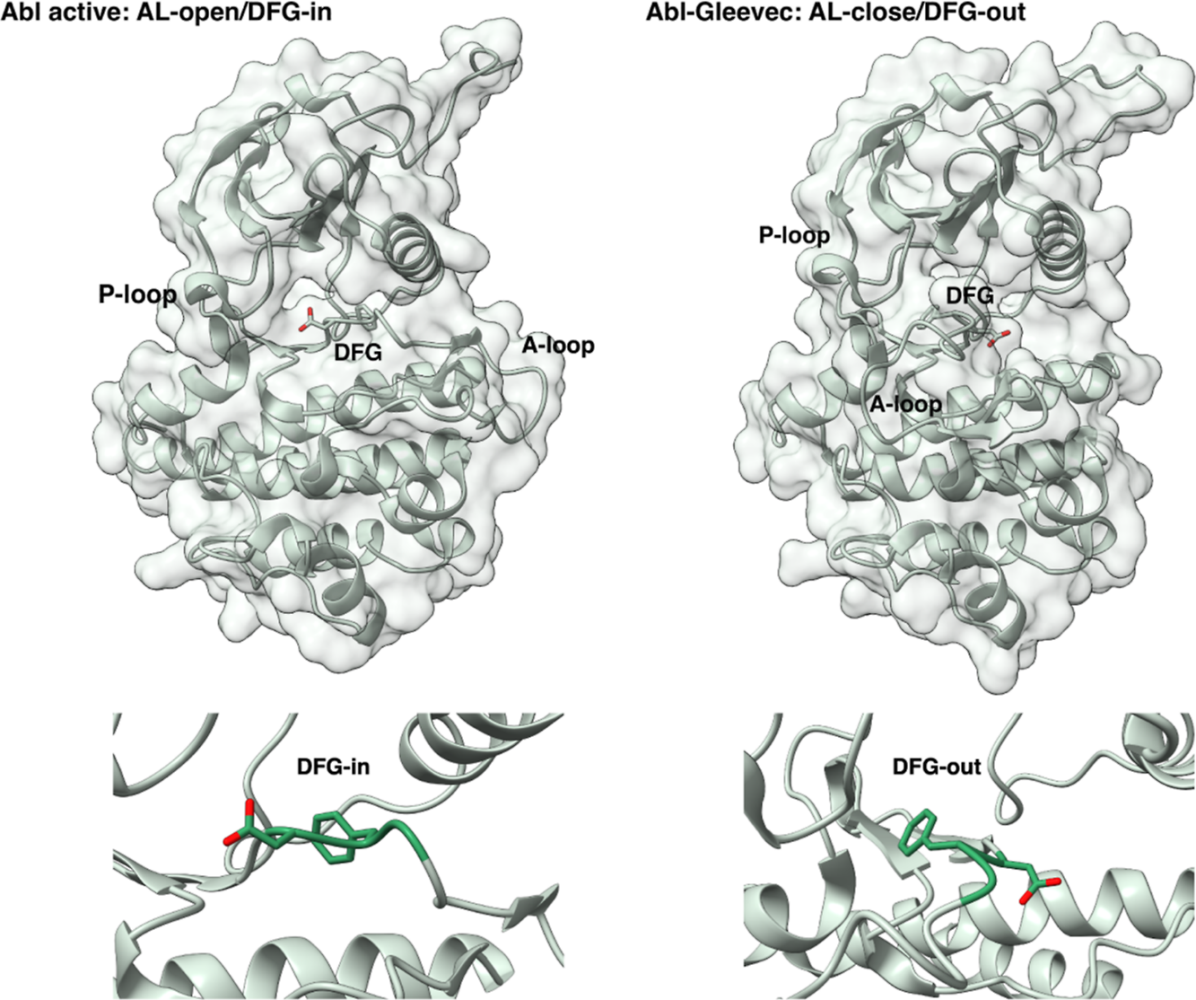
(Top) X-ray crystallographic structures of the c-Abl kinase (left) in an active state (A-loop in the open conformation and DFG-flip in the “in” conformation, PDB ID:2F4J) and (right) in an inhibited state, in complex with the ligand Gleevec (A-loop in the close conformation and DFG-flip in the “out” conformation, PDB ID:1IEP). The smaller amino-terminal part is the upper lobe, while the lower lobe represents the carboxyl-terminal part. The catalytic site lies in a cleft between the two lobes, and the relative orientations of the two lobes open or close the cleft. The activation loop adopts different conformations in the active/inactive states and it begins with the DFG sequence. (Bottom) Focus on the DFG motif (left) in the “in” conformation and (right) in the “out” conformation.

The binding mechanism and selectivity of Abl are strongly influenced by two loops: the activation-loop (residues 384–403) and the glycine-rich loop (residues 248–257). The activation loop (A-loop) is integral to the structure and function of the catalytic active site and begins with a highly conserved DFG (Asp-Phe-Gly) sequence that plays a crucial role in determining the active or inactive state of the receptor. When the kinase is inactive, the A-loop adopts a compact conformation, while when the enzyme is active, the A-loop is in a more extended conformation. The glycine-rich loop in the N-lobe, also known as the phosphate-binding loop (P-loop), primarily facilitates binding of the natural ATP ligand through hydrophobic interactions. Another pivotal amino acid residue is the gatekeeper Thr338, which regulates access to the active site.

To model the Abl–Gleevec complex, we started from the X-ray structure with PDB code 1IEP (chain A only), where the Abl A-loop in the carboxy-terminal lobe is in the closed conformation, with the catalytic motif in the DFG-out state. The ligand binds in the cleft between the amino- and carboxy-lobes of the kinase. In this inhibitor-bound state, two conserved residues, Leu248 and Val256, play crucial roles by providing key interactions with the inhibitor. When Gleevec binds in the binding site, its amide group acts as an anchor by interacting with adjacent Glutamate and Aspartate residues, helping to orient properly the ligand within the pocket. Additionally, the NH group of the secondary amine of Gleevec interacts with the side chain of the gatekeeper residue.

Abl was modeled with the AMBER99SB force field,^47^ while Gleevec using the General Amber Force Field (GAFF)^48^ and AM1-BCC point charges.^49^ The protonation states of amino acid residues were assigned based on their ionization states at physiological pH, assuming standard p*K*_a_ values. Additionally, the piperazine group of Gleevec is known to be protonated within the Abl pocket to facilitate interactions with surrounding residues. Thus, a positive charge was added to the outermost *N*-methyl piperazine nitrogen, in accordance with refs 6 and 50 . Solvation was carried out using the TIP3P water model^51^ within a cubic box with sides of 1.5 nm, large enough to ensure complete dissociation of the ligand from the protein pocket. To achieve physiological salt concentration (0.15 M) and neutralization, we introduced sodium and chloride ions. The fully solvated protein–ligand system then underwent energy minimization and equilibration, following the procedure already detailed in ref 2.

The topology and starting structure for the PreQ1 riboswitch in complex with the two ligands were taken from a recent work by Wang et al.^23^ In this work, the RNA-ligand complexes were modeled from the X-ray crystallographic structures with PDB codes 6E1W and 6E1U for the cognate and synthetic ligand, respectively. Ligands were parametrized according to the GAFF force field and AM1-BCC charges, considering a total charge of +1, due to the presence of a quaternary nitrogen in both. The systems were then solvated with TIP4P-D water model,^52^ a four-site model commonly employed in conjunction with the DESRES force field for RNA.^53^ Charges were neutralized with sodium and chloride ions, using a concentration of 0.15 M. The solvated systems were then equilibrated in two steps following ref 23.

In addition, we further reparameterized the cognate ligand with a more accurate representation for the charges via Restrained Electrostatic Potential (RESP) charges^54^ and optimized dihedral parameters, obtained via the PlayMolecule web server.^55^ Specifically, RESP charges were derived via quantum mechanics using the 6-311++G** basis set at the wB97X-D level of theory, while optimization of dihedral angle parameters was performed via the xTB method.^56^ As the starting X-ray structure (PDB code 6E1W) does not have hydrogen atoms, the cognate ligand can be bound to the RNA receptor in two possible tautomeric states. Consequently, an additional complex was constructed for the cognate ligand, starting from the same X-ray structure, but with a different ligand protonation state, representing the alternative tautomeric form of the ligand.

## Results and Discussion

In this study, we address the estimation of binding free energy (with a previously defined methodology^2^) in challenging biological systems of pharmaceutical relevance, namely a complex protein–ligand system together with the emerging class of RNA and their ligand partners. As test cases, we take the drug Gleevec binding to the Abl-tyrosine kinase and the preQ1 RNA riboswitch in complex with two ligands. Both these systems present remarkable challenges in terms of size and conformational flexibility. In the following sections, we discuss the results for these two test cases.

### Abl–Gleevec: Ligand Flexibility and Large Conformational Rearrangements

The complex formed by the Abl-tyrosine kinase and its potent inhibitor Gleevec holds significance not only from a therapeutic perspective but also due to its complexity, particularly due to the flexibility and size of Gleevec and the large rearrangements that Abl must undergo. Gleevec is a marketed drug widely used for cancer treatment that exhibits remarkable inhibitory activity for the Abl-tyrosine kinase (experimental Δ*F*_b_ = −10.9 kcal/mol^57^).

In a previous study,^2^ we applied our nonequilibrium protocol to investigate the Abl–Gleevec system, but we encountered significant challenges due to its large number of atoms and complex binding mechanism. In this work, we overcome such issues, obtaining a satisfactory binding free energy estimate for the Abl–Gleevec complex. Moreover, we streamline the computational procedure required to expand our protocol applicability to larger, pharmaceutically relevant biomolecular systems (with complex structure and dynamics), using Abl–Gleevec as relevant prototype in this class.

### Path Definition and PCVs for Abl–Gleevec

A proper parametrization of PCVs can help minimizing the dissipated work generated during nonequilibrium SMD simulations. This aspect is critical since high dissipated work (i.e., heat production) can hamper the convergence of binding free energy estimates. As we demonstrated in a recent work,^3^ improved PCVs can be obtained by introducing in the reference path, along with ligand atoms, other degrees of freedom of the system relevant to the process. This is mainly reflected in the *S* PCV definition, as these additional degrees of freedom will be pulled by the SMD bias. To improve the Abl–Gleevec PCV definition, we visually inspected the binding trajectories, since binding (and not unbinding) was the critical event in terms of dissipated work. Binding simulations revealed that the rapid pull of the ligand into the binding pocket did not allow sufficient time for the pocket to adapt. As a result, the Abl binding pocket maintained a predominantly closed conformation as the ligand approached, leading to significant dissipated work.

Our analysis aligns with other studies,^6,58,59^ which describe Gleevec binding to the apo-kinase as an extremely complex process involving a hybrid mechanism of conformational selection and induced fit. Moreover, the substantial conformational changes of the activation and glycine-rich loop must be taken into account to allow the binding pocket of Abl to adopt its previously identified, unusual tunnel-like shape.^50^

Consequently, the opening and closing of the Abl pocket during the binding and unbinding simulations is a crucial degree of freedom in the overall process. To properly include this conformational rearrangement during SMD simulations, heavy atoms of the binding pocket (within 6 Å from the ligand in the binding pose) were incorporated into the reference path. Thus, during binding simulations, the SMD bias potential not only pulls the ligand toward the binding pocket, but also guides structural rearrangements within the protein binding site. This refined path is anticipated to reduce the dissipated Jarzynski work, improving the convergence of binding free energy estimates. In particular, for binding simulations, this optimized path is expected to facilitate the conformational changes required in the target, thereby helping the binding mechanism.

Additionally, to define an optimal path, the exit trajectory of the ligand (its main axis) must be as orthogonal as possible to the protein surface during the unbinding event, especially for proteins with solvent-exposed binding pockets such as Abl. Moreover, for large and charged ligands like Gleevec, the conventional solvation shell definition used for smaller ligands is inadequate for accurately defining the unbound state. In the final configuration of the reference path, the ligand must be positioned at least 15 Å away from the protein surface, corresponding to more than three solvation shells, to avoid relevant electrostatic interactions in the unbound state (see Supporting Information section “Additional results for Abl-Gleevec”). Finally, the reference path must include all relevant intermediate metastable states involved during the binding event.

As anticipated, multiple ABMD simulations were performed to generate the initial trajectory to define the reference path and the PCVs. Longer ABMD simulations can promote a gentler, more realistic and spontaneous unbinding process. For this reason, we followed the same setup as in ref 2, except that now we performed several 20 ns ABMD runs (instead of 10 ns), simulating the unbinding of Gleevec from Abl, until a separation of at least 15 Å between the center of masses of the ligand and the binding pocket is reached. From these simulations, we selected the guess path based on the observed frequency of the mechanism, as well as the orthogonality of the ligand to the protein surface during unbinding. For each system studied, only a single predominant path is observed. This reference path was then refined using the previously mentioned path algorithms, resulting in an optimized path of 42 equidistant molecular configurations of both the ligand and pocket heavy atoms within 6 Å from the ligand in the bound pose, for a total of 51 residues. Among the residues selected to refine the path definition, we ensured the inclusion of key residues critical to protein rearrangements, as identified in the literature and confirmed by our ABMD trajectories. These include the DFG motif (Asp381, Phe382, Gly383), the first residue of the activation loop (Leu384), and nearly all residues of the glycine-rich loop. In particular, we included two conserved residues of the glycine-rich loop, Leu248 and Val256, which consistently interact with Gleevec in all bound conformations and remain in contact during the unbinding process, until Gleevec reaches its fully solvated state.

The main relevant configurations of the determined reference pathway are shown in Figure 3. The path starts from the bound pose found in the X-ray structure of the complex. Here, the inhibitor’s pyridine and pyrimidine rings occupy the binding site where the natural ligand binds, while the remaining portion of the inhibitor extends deeper into the hydrophobic core of the kinase, stabilizing it in its inactive conformation. Then, in this pathway, the significant rearrangements of the P-loop and the C-helix allow the unbinding of Gleevec, accompanied by movements of the A-loop. Gleevec unbinds orthogonally to the cleft between the N-terminal lobe and the C-terminal lobe, reaching the unbound state in the bulk. These observations are in agreement with the reported binding mechanism of Gleevec to Abl.^4,6^ However, capturing the complete conformational rearrangements associated with the transition of Abl from its inactive to active state is far from trivial and would require plain MD simulations on the microsecond time scale.^6^ As a result, the reference path induced by our fast ABMD simulations does not include the flip of the DFG-motif in the A-loop nor does it fully capture its extensive rearrangement, which involves a rotation of the A-loop that results in the displacements of up to 35 Å for central residues.^4^ Note that our goal here is to apply a standard procedure and not customize systematically the protocol for each system; the outcome is that our protocol does not capture completely the activation process, hence it represents an approximate coarse view of the binding event (see later for a further discussion).

**Figure 3:**
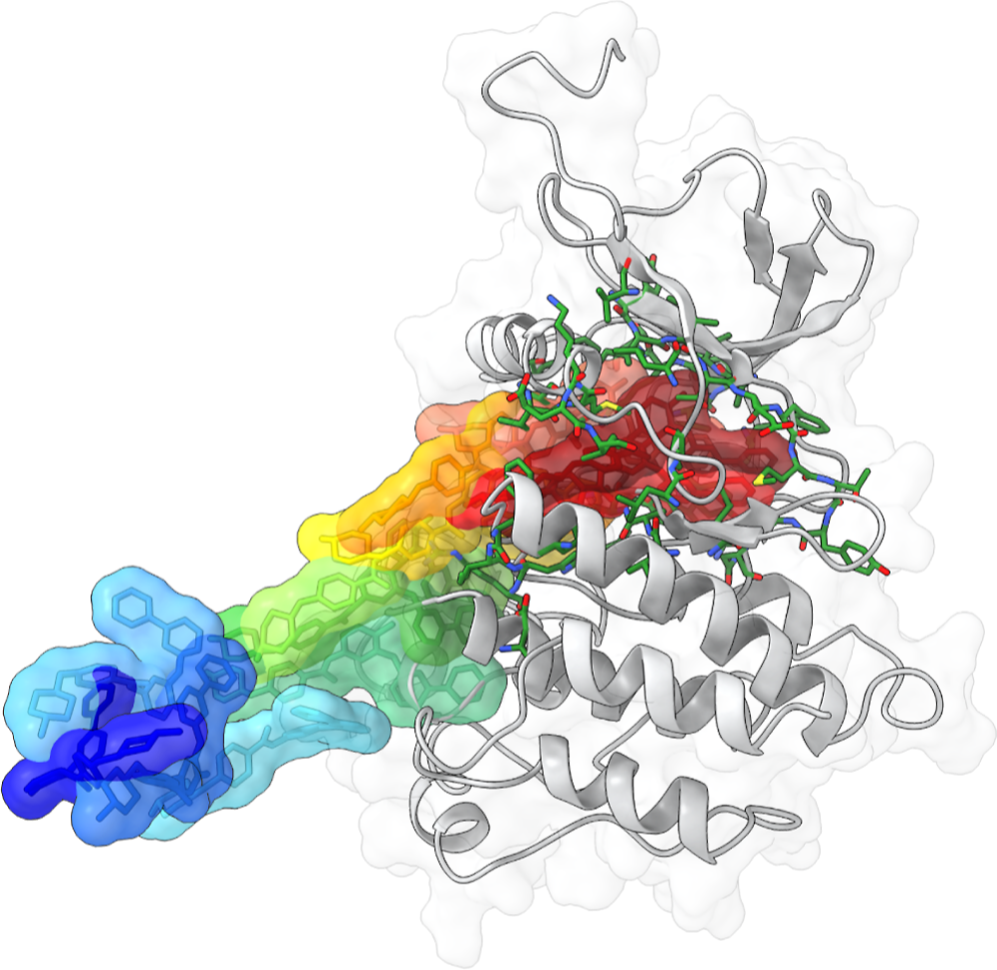
Schematic representation of the reference path of the Abl–Gleevec complex. Abl kinase is shown in gray in a ribbon representation, with the pocket atoms included in the reference path in green and licorice. The surfaces of the path configurations of Gleevec are represented from dark red for the bound state to dark blue for the unbound state (for clarity, only the most relevant configurations are displayed).

Finally, to minimize boundary effects of PCVs that could distort the FES profile, fictitious configurations generated with short SMD runs were added at both ends of the reference path. Despite being physically unreachable during simulations, these configurations help to improve the sampling of the true bound and unbound states.^3^

### Binding Free Energy Estimates

Using the newly defined reference path for PCV-based sampling via SMD simulations, we performed 30 production runs for both binding and unbinding events. Additionally, we carried out the simulations for two time lengths, 100 and 200 ns (i.e., for two different pulling speeds, since we performed simulations with a restraint at constant velocity). To understand whether these SMD simulation times were sufficient to reach convergence of the estimates, we performed a statistical analysis assessing convergence as a function of the number of replicas.

We calculated *W*_J_ values for the system, obtaining the work profiles over the simulation times reported in Supporting Information (Figure S3). These profiles demonstrate a reduction in the dissipated work during SMD simulations compared to the previously obtained ones (ref 2 Figure 8), resulting from the inclusion of pocket atoms in the reference path. Furthermore, the similarity of the work curves at 100 and 200 ns suggests that 100 ns already provides a sufficiently gentle pulling speed to reach convergence.

Using our procedure based on the CFT estimator, we reconstructed the FESs along *S*(*x*), shown in Figure 4. The error details for each point of the FES are provided in the Supporting Information in Figure S4. The 100 ns free energy profile reveals a higher activation energy barrier for binding, probably because faster SMD simulations are more affected by kinetics, resulting in elevated barriers in the free energy profile. Nonetheless, the similarity between the free energy profiles (as for work curves) obtained at both pulling speeds indicates that 100 ns is sufficient to achieve thermodynamically (not kinetically) accurate results.

**Figure 4:**
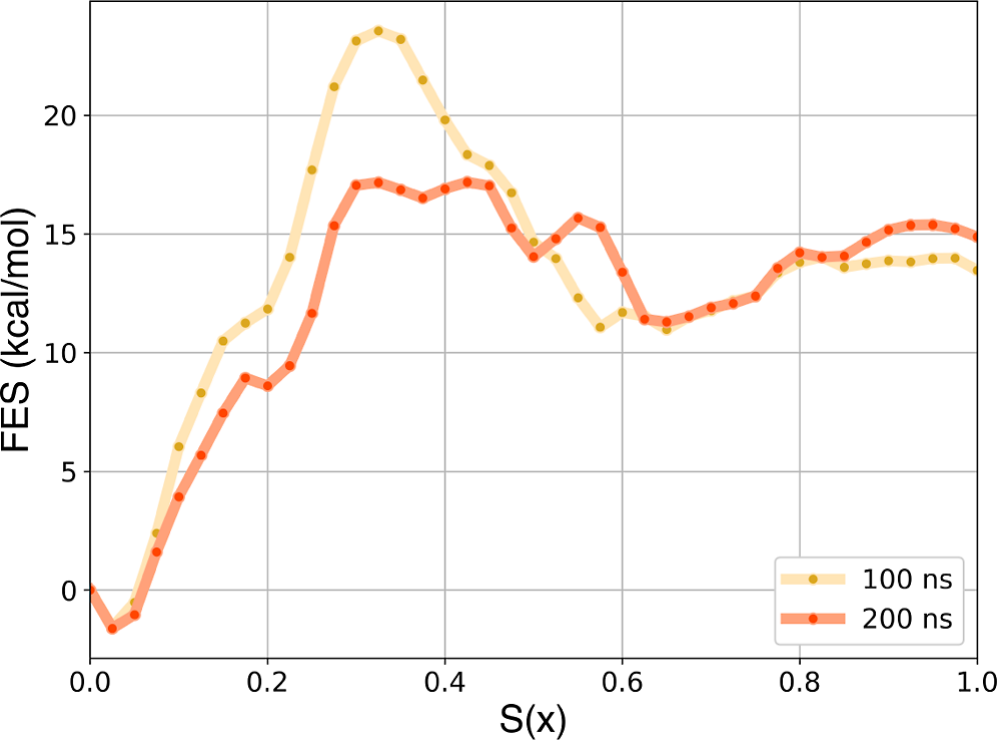
Free energy profiles along *S*(*x*) obtained by applying CFT to SMD of 100 and 200 ns with the refined PCVs for Abl–Gleevec.

To calculate the binding free energy from the FESs, we computed the ratio of the bound and unbound partition functions, selecting the 18th configuration as the molecular structure distinguishing the bound and unbound states. After applying the standard volume correction (amounting to −1.4 kcal/mol), we obtained standard binding free energies comparable to the experimental reference of −10.9 kcal/mol. The CFT-derived estimates from 100 and 200 ns SMD simulations are −12.5 ± 1 kcal/mol and −13.0 ± 2 kcal/mol, respectively, in fair agreement with the experimental value. The results show that the inclusion of the protein pocket degrees of freedom leads to a reduction of the dissipated work, improving the convergence and precision of binding free energy estimates.

Finally, we evaluated the necessity of the new path criteria and the improvements they bring to the reference path definition with an independent sampling approach. Specifically, we performed Well-Tempered MetaD simulations using With the refined reference path, Gleevec is able to move further away from the protein, sampling the bulk state more extensively. This qualitative observation underscores the importance of the newly introduced criteria. The FES, *S*(*x*) profile, and hills deposition from the Well-Tempered MetaD simulations are available in the Supporting Information.

### Conformational Rearrangements and DFG-Flip Transition

Experimental and computational studies have highlighted the crucial role of the DFG-motif in Gleevec inhibitory action for Abl.^6,50,60^ As reported by Schindler et al.,^61^ in order for Gleevec to bind to the Abl pocket, the DFG motif needs to undergo a conformational rearrangement from an active to an inactive state, characterized by a 180° rotation of the DFG motif called DFG-flip.

Consequently, in the Abl–Gleevec complex, the DFG-motif is in the out inactive conformation, while the activation loop adopts a closed conformation, preventing the binding of the natural substrate. Moreover, the glycine-rich loop is folded in a kinked conformation toward the binding pocket, facilitating favorable interactions with the bound ligand. The glycine-rich loop conformation is stabilized by a specific hydrogen bond between Tyr253 and Asn322.

Various computational methods have been employed to assess the contribution of the DFG-flip to the overall binding free energy of the complex Abl–Gleevec. As reported by the Roux’s group in ref 50, the DFG-flip resulted to be a relevant contribution to the binding free energy, amounting to 1.4 kcal/mol.

However, during our 200 ns simulations, the DFG-flip was not observed. Conformational rearrangements of both the P-loop and A-loop occur during simulations, while the DFG motif remains stable over time (Figure S5).

Although our reference path included the residues of the DFG-motif, the rearrangement sampled in the ABMD simulation does not reflect a transition of this motif from a closed to an open configuration. To simulate the DFG-flip, a tailored collective variable or longer simulation time would be required. In ref 62, in order to observe this rearrangement, a 200 μs plain MD simulation was run. Consequently, during the 20 ns unbinding ABMD trajectory, obtained using an electrostatic-like CV between the ligand and pocket atoms, the DFG-motif retained an out conformation even when reaching the unbound state.

To qualitatively account for the relevant contribution to the free energy difference of the DFG-flip in our results, we can include in our standard binding free energy estimates a correction term due to Δ*F*_in→out_ = 1.4 kcal/mol, as computed in ref 50. If we account for this contribution, the CFT estimates for 100 and 200 ns simulations become −11.1 and −11.6 kcal/mol respectively, hence providing a correction in the correct direction with respect to the experimental value.

### Riboswitch-Ligand: Receptor Flexibility and Conformational Rearrangements

Having established the methodology for protein systems, we explored the applicability to another challenging class of biomolecules, namely RNAs.

### Path Definition and PCVs for RNA-Ligand Systems

As demonstrated with complex proteins, optimal parametrization of PCVs minimizes dissipated work during nonequilibrium SMD simulations, thereby improving the convergence of free energy estimations. Similarly, defining an optimized and plausible unbinding path is of critical importance also for RNA-ligand systems.

A total of 10 ABMD simulations, each lasting 10 ns, were run to generate the initial trajectories for each of the two RNA-ligand complexes in TIP4P-D water model. These trajectories were guided by a collective variable based on the Debye–Hückel interaction energy between the highly negative charges of RNA and the positive charge of the ligands. Particularly, they were guided toward a collective variable value of zero, which represents complete ligand unbinding. The trajectories were then cut when the distance between the ligand and the RNA exceeded 20 Å, ensuring a realistic unbound state. In these simulations, both ligands dissociated through a solvent-exposed pathway located between loop 2 (residues U12-U13-A14-U15-A16-C17) and stem 1 (residues U9-A10-G11 and C31-U32-A33-A34), as illustrated in Figure S9. This consistent unbinding mechanism involved structural rearrangements of loop 2 and stem 2, opening up the binding pocket to facilitate ligand exit.

The Debye–Hückel interaction energy during the ABMD simulations is reported in Figure S6. These plots demonstrate the reduction of the interaction energy as the ligands unbind, with final values of 1.73 and 1.14 kcal/mol for the cognate and synthetic ligands, respectively.

A key distinction between protein and RNA systems lies in the greater structural flexibility exhibited by RNA.^16,17,63,64^ Consequently, defining a reference pathway for PCVs for RNA-ligand complexes necessitated specific adjustments.

An implicit assumption of the PCV calculation is the inherent reliability of the optimal-alignment mean-square deviation between the configurations of the reference path and the instantaneous configuration during the simulation. For protein–ligand complexes, the Cα of the protein were used to align the instantaneous configuration to the reference configurations. However, because of the highly flexible nature of RNA, identifying suitable atoms for alignment is less trivial, yet necessary to define a correct alignment and PCV values. To define suitable atoms, we performed three unbiased 100 ns MD simulations and aggregated the data to calculate residue-wise root-mean-square fluctuations (RMSF) reported in Figure S7. We identified residues with an RMSF below a 1.8 Å threshold and we selected a subset of their atoms for the alignment, as shown in Figure S8. Specifically, of those residues we used the *P* and *C*1′ carbon atoms of the RNA backbone for alignment. Notably, this is also conceptually consistent with the alignment selection used for the protein system.

Additionally, the selection of atoms of the RNA-ligand system to be included in the reference pathway necessitates careful consideration, particularly crucial in RNAs complexes, since the ligand (un)­binding process is associated by rearrangements in stem 1. This region forms an intricate hydrogen-bonding network with the cognate ligand and adopts specific conformations of residue C15 to accommodate the bulkier synthetic ligand within the binding site (Figure 5).^24^

**Figure 5:**
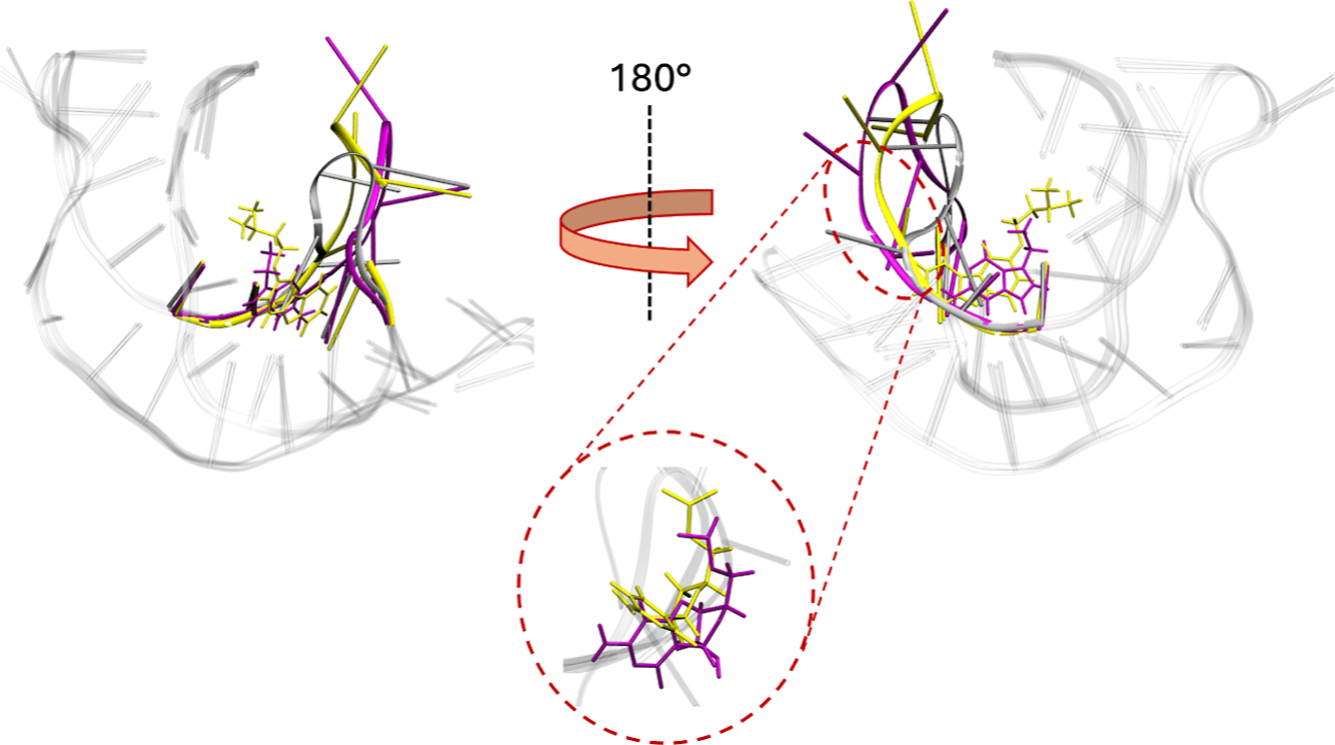
Structural superposition of the apo (white), cognate ligand-bound (purple), and synthetic ligand-bound (yellow) states of the receptor, highlighting the conformational rearrangement of loop 1. A close-up view, on the left, reveals the distinct orientations of residue C15 induced by the different ligands.

Therefore, to account for these structural rearrangements, the path definition included RNA atoms within 6 Å of the ligand in the ABMD unbinding trajectories. Specifically, *N*1, *C*8, and *C*1′ atoms were included for purines, while *C*6, *N*3, and *C*1′ atoms were considered for pyrimidines (Figure S8). This resulted in an optimized unbinding reference path for PCVs consisting of approximately 35 equidistant conformations for each RNA-ligand system (Figure 6).

**Figure 6:**
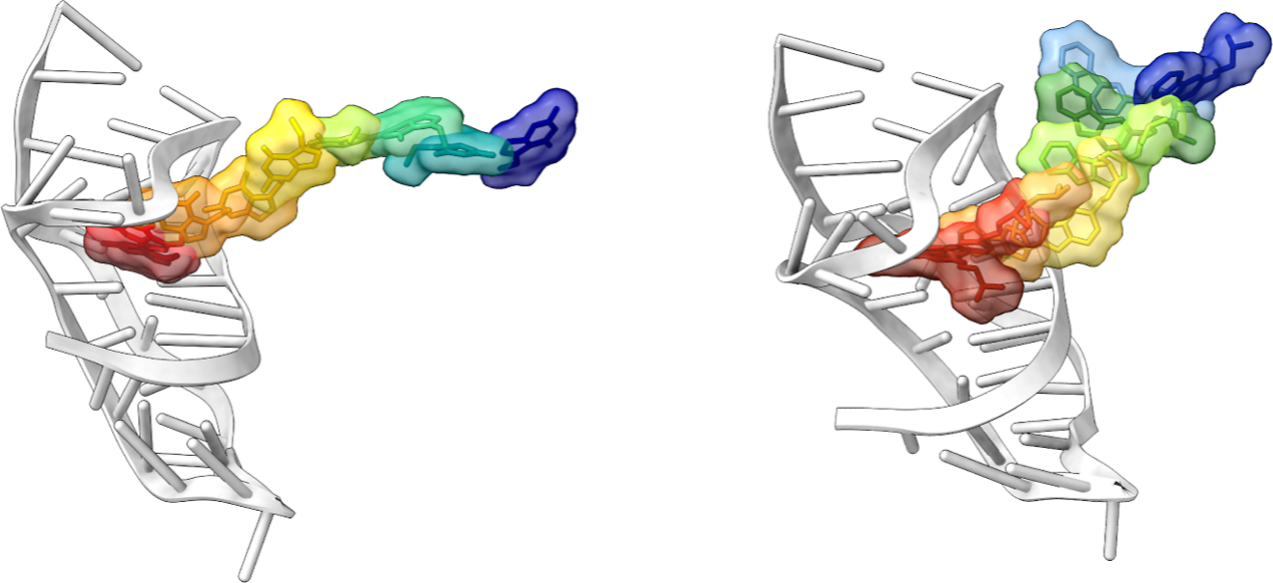
Schematic representation of the reference paths of riboswitch-preQ1 in complex with (left) the cognate ligand and (right) the synthetic ligand. The RNA is shown in light gray. The surfaces of the ligand configurations are colored from dark red for to the bound state to dark blue for the unbound state (for clarity, only the most relevant configurations are displayed).

### Binding Free Energy Estimates

After the path optimization we followed the same protocol used for the Abl–Gleevec complex, performing 30 replicates of SMD binding and unbinding 100 ns simulations. During the SMD simulations, we calculated the Jarzynski work *W*_J_, obtaining the work profiles over the simulation time, as reported in Figure S11. The unbinding simulations of the cognate ligand (Figure S11A) show a higher dissipation in disrupting the hydrogen network with the pocket compared to those of the synthetic ligand. When the ligand reaches the unbound state and is fully solvated, the work profile reaches a plateau.

In contrast, the dissipation during binding simulations required to reach the binding pose (Figure S11B) is fairly similar for both ligands. Different replicas exhibit a different dissipation, reflecting the complexity of the ligand association process. Nevertheless, the work dissipation is limited for both processes, demonstrating the suitability of the criteria to define an optimal reference path for RNA-ligand systems.^3^

Applying our protocol based on the bidirectional CFT estimator, we reconstructed the FESs along *S*(*x*) for both ligands in TIP4P-D water model (Figure 7). To calculate the binding free energy from the FESs, we selected a discriminative conformation corresponding to *S*(*x*) values of 16 and 14 for the cognate and synthetic ligands, respectively. Standard binding free energies were obtained by computing the ratio of the bound and unbound partition functions and adding the standard volume correction. This correction contributes 0.6 kcal/mol for the complex with the natural ligand and 0.2 kcal/mol for that with the synthetic ligand. These results were compared with experimental affinity data from recent literature.^23,24^ The estimated standard binding free energy (Δ*F*°) for the RNA-synthetic ligand complex is −5.6 ± 1 kcal/mol, slightly deviating from the experimental value of −7.9 kcal/mol. For the RNA-cognate ligand complex, Δ*F*° is estimated to be −17.2 ± 1 kcal/mol, with a more pronounced discrepancy compared with the experimental value of −10.9 kcal/mol.

**Figure 7:**
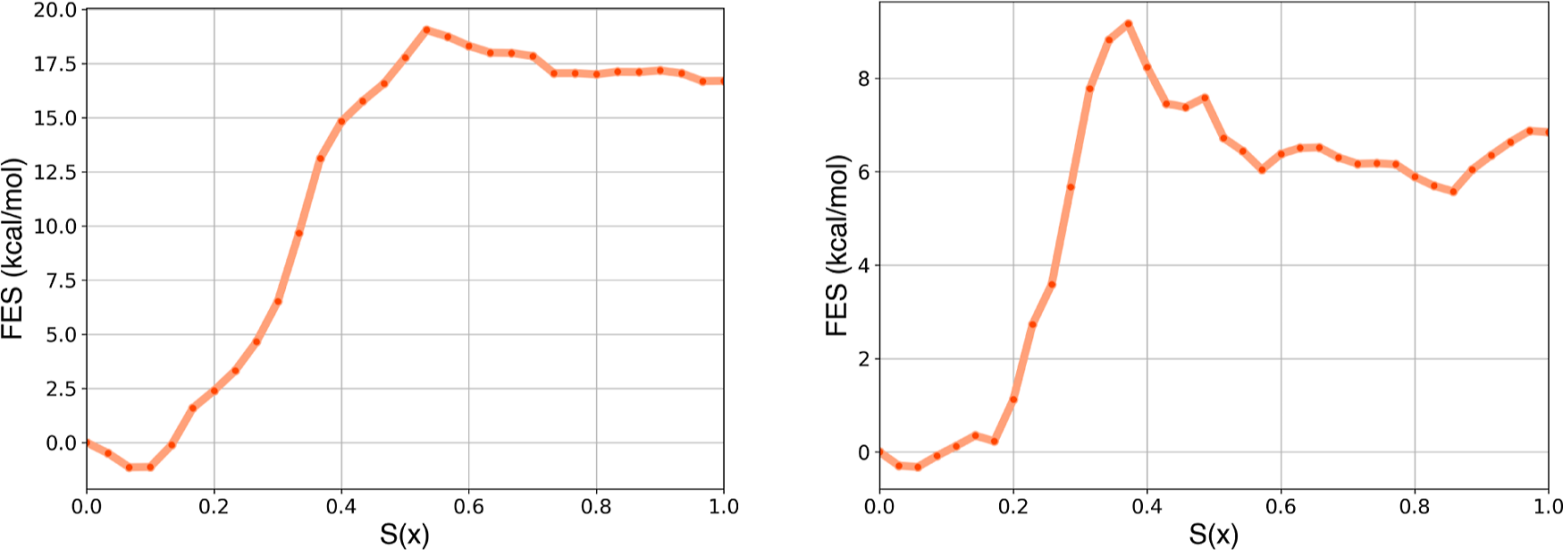
Free energy profiles along *S*(*x*) obtained by applying CFT to SMD (simulation time of 100 ns in TIP4P-D) for the cognate (A) and synthetic (B) ligands.

While the free energy profile along *S*(*x*) determined with the CFT for the cognate ligand appears reasonable, the ones obtained for binding simulations using the Jarzynski equality revealed a scattered unexpected trend (Figure S13A). This trend may be linked to the lower capacity of the Jarzynski estimator to mitigate the dissipative work. This high dissipation likely contributes to the error in the binding free energy estimate for this ligand.

### Water Model Effect on Binding Free Energy Estimates

Hitherto, all simulations for RNA-ligand complexes were performed using the TIP4P-D water model. However, recent findings^3^ suggest that the four-site model may not be the most optimal choice to achieve good agreement between experimental results and calculations in nonequilibrium simulations. In fact, the TIP3P model might be less affected by work dissipation, leading to estimation with lower bias. Although TIP3P is neither the most recent water model nor the ideal choice for RNA, it may prove to be the most practical option for nonequilibrium simulations.

Therefore, to explore the potential impact of the water model on the binding free energy estimates in our nonequilibrium SMD simulations, we repeated all simulations using the less dissipative TIP3P model,^65^ while maintaining the same reference path (as performed in ref 3).

The systems were solvated with TIP3P waters, equilibrated and, subsequently, SMD simulations were conducted with the same setup employed before. For both ligand, the general trend of the work curves with TIP3P water (Figure S12) revealed no significant differences compared to the TIP4P-D results (Figure S11). However, for the synthetic ligand, there is a non-negligible effect of the water model on the work dissipation. As seen for proteins, also for the RNA-ligand systems investigated here, the TIP3P model is shown to be less affected by dissipation than the TIP4P-D model.

A visual inspection of the simulations revealed conformational differences of the RNA in the bound state with TIP3P water compared with TIP4P-D water. Simulations with the TIP4P-D model showed difficulties in tightly following the reference pathway in the last stages (i.e., when reaching the bound state). Indeed, in these simulations the stem 1 is unable to assume the exact conformation of the X-ray crystallographic structure.

Consequently, for the synthetic ligand the CFT-derived FES (Figure S14B) and the resulting binding free energy obtained with TIP3P deviate from those estimated with TIP4P-D. The estimated Δ*F*° for the RNA-synthetic ligand using the TIP3P water model is −8.7 ± 0.7 kcal/mol (discriminating frame: 14th molecular configuration of the reference path), demonstrating better agreement with the reference experimental value (−7.9 kcal/mol) compared to the TIP4P-D model, which yielded a value of −5.6 ± 1 kcal/mol. In contrast, for the cognate ligand, the FES (Figure 8A) and the standard binding free energy value obtained with TIP3P were rather similar to those obtained with TIP4P-D. The Δ*F*° for the RNA-cognate ligand estimated using TIP3P is −18.5 ± 3 kcal/mol, still deviating from the reference experimental value of −10.9 kcal/mol.

**Figure 8:**
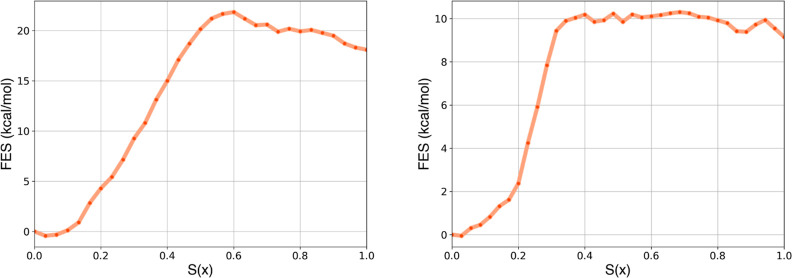
Free energy profiles along *S*(*x*) obtained by applying CFT to SMD (simulation time of 100 ns in TIP3P) for the cognate (A) and synthetic (B) ligands.

The results for the synthetic ligand highlight the significant impact of the chosen water model on the accuracy of nonequilibrium free energy estimations. While simulations employing the TIP4P-D water model underestimated the binding free energy (Δ*F*), those using the TIP3P water model yielded values that fell within the experimental error range.

Nevertheless, for the cognate ligand, also with the less viscose TIP3P model, the SMD results still display high discrepancy from the reference experimental data.

To further assess the reliability of our nonequilibrium method on RNA-ligand systems, we also calculated the binding free energy with an independent sampling approach. This analysis is rather interesting because Well-Tempered MetaD does not rely on the Jarzynski work for the free energy estimation. On the other hand, the nonequilibrium approaches based on the CFT and Jarzynski estimators are heavily dependent on this parameter.

Thus, we performed Well-Tempered MetaD simulation with PCVs, using the previously generated reference pathways and the TIP4P-D model. The Well-Tempered MetaD simulations were able to exhaustively sample the *S*(*x*) PCV, allowing to observe multiple (un)­binding events in each simulation, as both ligands were able to transition multiple times between the bound and the unbound state (Figure S19). Interestingly, the cognate ligand resides in the bound state for a significant fraction of the total simulation time. This is because the cognate ligand forms a higher number of interactions with RNA in the bound state. After reconstructing the FES (Figure S20), we estimate the binding free energy. The results obtained from the Well-Tempered MetaD simulations are reported in Table S2 and compared with the SMD results.

The Well-Tempered MetaD results are different from those obtained with the SMD simulations using TIP4P-D. However, the results obtained with Well-Tempered MetaD exhibit better agreement with the SMD simulations with the TIP3P water model (see Table S3). This suggests that models like TIP3P may be more suitable for reducing dissipative work in SMD simulations, while more accurate (although more expensive) models such as TIP4P-D may be more appropriate for Well-Tempered MetaD simulations.

### Possible Source of Errors for the Cognate Ligand

The binding free energy values obtained for the cognate ligand deviates remarkably from the reference experimental value. In both the SMD and Well-Tempered MetaD simulations, the cognate ligand appears to have a higher binding affinity compared to the experimental one. Differently, the calculated binding affinity for the synthetic ligand is in agreement with the experimental value.

This discrepancy could arise from the fact that a correct estimation of the binding affinity to the RNA target is more difficult for the cognate ligand compared to the synthetic one, as the cognate ligand is more strongly bound to the target and has an experimentally reported higher affinity. Particularly, the crystallographic poses of the two ligands revealed how the binding mode of the cognate ligand is driven by a higher number of interactions with the RNA target. Both ligands form a comparable amount of stacking interactions with RNA, however, the cognate ligand establishes 7 hydrogen bonds with the target, while only 2 are formed with the synthetic ligand (see Figures 9B and S10). Consequently, the vigorous interactions between RNA and the cognate ligand introduced challenges in accurately estimating the binding free energy for this complex. Here, we investigated potential sources of discrepancies between our results for the cognate ligand and the experimental affinity.

**Figure 9:**
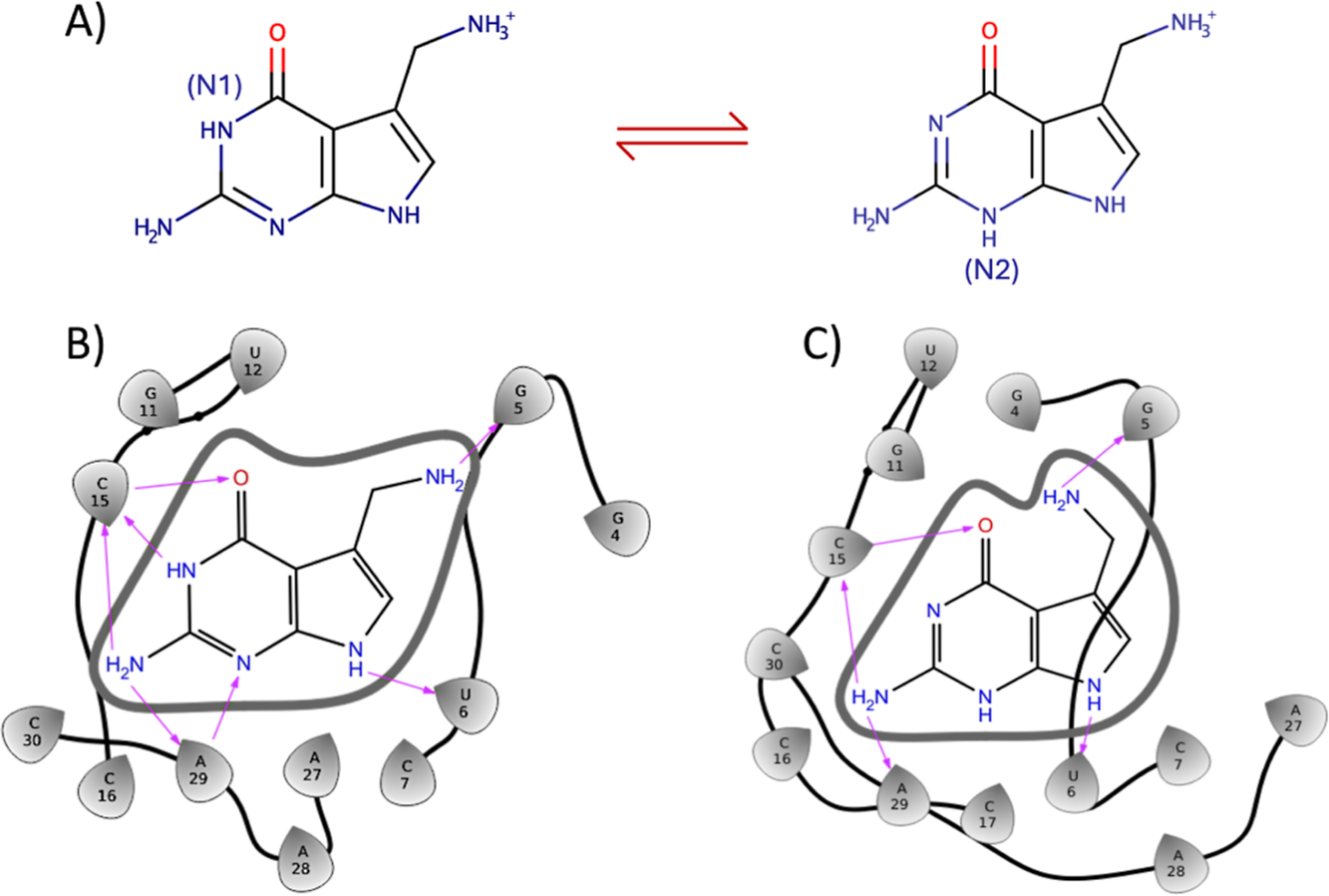
(A) Structures of the two tautomeric forms of the cognate ligand: N1 (left) and N2 (right). (B) Hydrogen bonding networks formed between the RNA target and the cognate ligand in the tautometric form N1 and (C) in the tautomeric form N2. The two tautomeric forms establish a different hydrogen networking with the RNA target.

A potential source of error could be due to the inaccuracy of force fields for RNA-ligand simulations. However, macromolecule force fields have improved significantly over the years, becoming progressively more reliable and increasingly capable of quantitative prediction of experimental observables. While this is true for macromolecular species such as proteins and RNAs, small molecule parametrization is still lagging behind. This is mainly due to the heterogeneity and wide size of the chemical space associated with the small organic molecules that can potentially be designed. In particular, inaccuracies in charge and dihedral angle parameters can impact remarkably simulations results.^66^ In this respect, nowadays it is rather standard to use the AM1-BCC method to assign charges to ligands for MD simulations. This usually results in a high-quality description of electrostatic properties of ligands in most cases. However, AM1-BCC charges may be insufficient in more complex scenarios. In these cases, resorting to a more accurate theory level, such as using charges derived from Density Functional Theory and the Restrained Electrostatic Potential (RESP) method, may be more appropriate.^54^ For the systems studied here, a large number of heteroatoms participate in the formation of hydrogen bond interactions with the RNA target in the bound state. Therefore, we improved the parametrization of the cognate ligand with RESP charges and optimized dihedral angles parameters. Then, we applied our protocol to generate again a guess unbinding pathway via ABMD simulations, and performed the production SMD runs. Interestingly, the preferred unbinding pathway, passing through loop 2 and stem 1, was consistent with the one observed using the previous ligand parametrization and was similar across all ABMD runs. Moreover, to assess the effect of the different water models in combination with the newly parametrized ligand, both the TIP4P-D and TIP3P water models were considered. The results are reported in Table S4.

The results obtained for the RESP parametrized cognate ligand are not strongly affected by the water model. Indeed, the binding free energies obtained in TIP3P and TIP4P-D solvent are fairly similar (within the statistical error), as for the results obtain with AM1-BCC charges. However, the results with RESP parameters are fairly consistent with the ones with the AM1-BCC parameters, both deviating from the experimental value. This may suggest that the charge model is not the major source of errors in this case.

Furthermore, to ensure consistency of our results and definitively exclude the parametrization procedure as a source of error, we reparametrized all ligands, i.e. both synthetic and cognate. Thus, instead of using topologies from ref 23, we reconstructed from scratch the topologies for all ligands, using the AM1-BCC charge model and GAFF (additional details provided in the Supporting Information). This provided consistent results, confirming the reproducibility of our findings and excluding the parametrization procedure as the source of discrepancy with experimental data.

Another potential source of inaccuracy for the cognate ligand may arise from the presence of different tautomeric states in solution.^67^ QM calculations (details in Supporting Information) revealed the possibility of having a different tautomeric form in solution. In particular, the cognate form considered hitherto, defined N1, can tautomerize into the form N2 by the shift of a proton from N1 (cognate N1) to N2 (cognate N2), as reported in Figure 9A. This alternative state N2 may be promoted in solution by the intramolecular hydrogen bond between the carbonyl and the protonated amine groups. Consequently, for completeness of our results, we decided to include the tautomeric state N2 in our simulation panel by repeating the entire pipeline with the cognate ligand in this alternative tautomeric form.

To obtain a comprehensive picture and compare with the other results, we conducted SMD simulations with the TIP4P-D and TIP3P water models and performed Well-Tempered MetaD simulations. The binding affinity calculated for the tautomeric form N2 is significantly lower than the one for the form N1. However, the results for the cognate ligand in the tautomeric form N2 show a remarkably improved agreement with the reference experimental data. Specifically, in the TIP4P-D simulations, the standard binding free energy is −13.2 ± 1 kcal/mol, while in TIP3P it is −11.9 ± 1 kcal/mol, in fair agreement with the experimental value of −10.9 kcal/mol (Table S4). Moreover, they remain statistically consistent with the independent results from the MetaD simulation in TIP4P-D water, which yielded −13.9 ± 1 kcal/mol. All the results for the cognate ligand are summarized in Figure 10.

**Figure 10:**
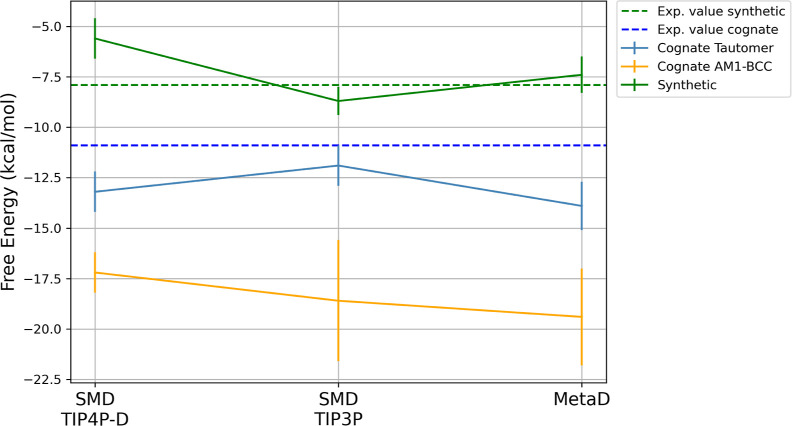
Binding free energies for all complexes tested. Dashed lines indicate experimental values (green: synthetic, blue: cognate). The *x*-axis represents the calculation method (SMD in TIP4P-D and TIP3P or MetaD), while the *y*-axis shows the corresponding binding free energy with associated errors. Ligands are color-coded: synthetic (green), cognate with AM1-BCC charges (orange) and cognate tautomer (light blue).

Notably, the tautomeric state N2 is a less expected form, as the cognate ligand N1 closely resembles the Guanine nucleobase with only minor modifications. Thanks to this similarity, the cognate ligand N1 is more likely to establish the expected hydrogen bonding network within the binding site, interacting with nucleobase C15 through their Watson–Crick edges. This is consistent with the behavior of Guanine in this specific three-dimensional arrangement (Figure 9B). As shown in Figure 9B,C, the cognate ligand in state N2 loses two hydrogen bond interactions with C15 and A29 compared to the state N1.

Furthermore, analysis of 500 ns plain MD simulations (two replicas) revealed distinct hydrogen bonding patterns for the two tautomers within the binding pocket over time. Specifically, in the N1 tautomer simulations, the number of hydrogen bonds remained stable at approximately seven, whereas in the N2 tautomer, the average number of hydrogen bonds was five. The corresponding hydrogen bond plots are provided in Supporting Information. These observations on the hydrogen bond network may help explain the lower binding affinity calculated for the N2 tautomeric form.

Finally, the presence of both tautomeric forms in solution could explain the discrepancy of the experimental affinity of the cognate ligand compared to our simulations results for the tautomer N1. As both tautomeric form coexist in solution, we may speculate that the experimental binding free energy is lower than the affinity of only the stronger binder tautomer N1, due to the contribution of the weaker binder tautomer N2.

## Conclusions

In this study, we addressed the binding free energy estimation in challenging biological systems of pharmaceutical relevance, using nonequilibrium MD simulations. In particular, we focused on the binding of the Gleevec drug to the Abl protein target, and of two ligands to the preQ1 RNA riboswitch. These complexes present inherent challenges given the realistic size of Abl–Gleevec, the conformational rearrangement of the Abl protein, and the marked flexibility of the RNA receptor. Building on recent insights,^3^ we optimized the criteria for the construction of the reference binding pathways for PCVs. In particular, we emphasized the inclusion of all degrees of freedom that are critical to the binding process in the reference pathway, such as both the ligand and the binding pocket atoms for the systems treated here. This was crucial to capture the intricate structural rearrangements in the receptor binding sites, relevant both in the Abl–Gleevec system, given the complexity of the binding mechanism, and in the riboswitch-ligand systems, due to the inherent structural flexibility of the RNA molecule. Upon construction of robust reference pathways for PCV-based MD simulations, we were able to estimate standard binding free energies for both the protein- and RNA-ligand systems considered. The estimation for Gleevec exhibited higher consistency with the experimental value, confirming the inherent difficulties of RNA system calculations. Interestingly, our investigation confirmed that using the less dissipative TIP3P water model is preferable in a nonequilibrium setting, resulting in estimates in better agreement with experiments than the four-point model TIP4P-D. Conversely, when used in conjunction with MetaD, the four-point model TIP4P-D provided results compatible with experiments. This observation aligns with our recent findings,^3^ demonstrating the greater sensitivity of nonequilibrium steered molecular dynamics to the kinetics of the systems, and highlighting the importance of selecting suitable water models depending on the employed simulative approach. Although equilibrium approaches are more traditionally applied in the field of free energy calculations,^68^ nonequilibrium methods still represent an open challenge and may present computational advantages. In particular, the main advantage of the proposed pipeline lies in its straightforward parallelization compared to methods such as MetaD. Despite the total simulation time required by nonequilibrium SMD being significantly longer than that of MetaD, all simulations can be executed in parallel. Therefore, by leveraging high-performance computing architectures, the time-to-solution is determined by the execution time of a single replica, i.e., 100 ns for the systems discussed in this work. The execution time of a single replica was selected as a trade-off between the single replica time and the number of replicas to achieve the fastest convergence with the fastest time-to-solution. Finally, the proposed workflow allows for a more seamless assessment of convergence compared to MetaD. Undoubtedly, the methodology presented here is more computationally demanding than established and cost-effective “end-point” methods. Nevertheless, in addition to yielding binding free energy estimates, our approach can provide mechanistic insights into the binding process and relevant intermediates, which can be extremely useful within drug design endeavors. This information can be particularly valuable for effective applications in pharmaceutical research.

## Supplementary Material



## Data Availability

All the data and scripts to reproduce our findings are available at https://gitlab.iit.it/hpc/FreeEnergyPath/. Some scripts and simulations require the BiKi Software. A temporary license can be requested for free to reproduce our findings.

## Notes

Additional results for Abl–Gleevec: Analysis of PCVs and Path definition, Work Profiles and FESs, DFG-flip contribution. Additional results for RNA-ligand systems: Debye–Hückel interaction energy profile, Analysis of PCV and Path definition, 3D structure of Riboswitch, Work Profiles and FESs. Well-Tempered MetaDynamics additional results: Well-Tempered MetaDynamics of Abl–Gleevec, Well-Tempered MetaDynamics of RNA-ligands. Possible source of errors for the cognate ligand - additional results: Additional parameters, Hydrogen bonds analysis (PDF)
