# Supplementary material for: Path-Based Nonequilibrium Binding Free Energy Estimation, from Protein–Ligand to RNA-Ligand Binding
Source: J Chem Inf Model. 2025 Jun 6;65(12):6057–72. doi: 10.1021/acs.jcim.5c00452 (PMC12199290; doi:10.1021/acs.jcim.5c00452)
Supplement: Supplementary file 1 [file ci5c00452_si_001.pdf]

Supporting Information for: Path-based nonequilibrium binding free energy estimation, from protein-ligand to RNA-ligand binding

Eleonora Serra,[†,‡,⊥] Alessia Ghidini,[¶,⊥] Riccardo Aguti,[†] Mattia Bernetti,[*,§,‡] Sergio Decherchi,[*,‖] and Andrea Cavalli[¶,‡]

†*Department of Pharmacy and Biotechnology (FaBiT), Alma Mater Studiorum - University of Bologna, via Belmeloro 6, 40126 Bologna, Italy*

‡*Computational & Chemical Biology, Fondazione Istituto Italiano di Tecnologia, via Morego 30, 16163 Genoa, Italy*

¶*Centre Européen de Calcul Atomique et Moléculaire (CECAM), Ecole Polytechnique Fédérale de Lausanne, 1015 Lausanne, Switzerland*

§*Department of Biomolecular Sciences, University of Urbino "Carlo Bo", Piazza Rinascimento 6, 61029 Urbino, Italy*

‖*Data Science and Computation Facility, Fondazione Istituto Italiano di Tecnologia, via Morego 30, 16163 Genoa, Italy*

⊥*These authors contributed equally to the work.*

E-mail: mattia.bernetti@uniurb.it; sergio.decherchi@iit.it

Additional results for Abl-Gleevec

In this section, additional information and results of Abl-Gleevec complex are reported.

Analysis of PCVs and Path definition

Building on the original path definition from Ref.,[1] we initially refined the reference path by incorporating binding pocket atoms within 6 Å of the ligand in the bound pose. Starting from the same ABMD unbinding trajectory of Ref.,[1] we simply reapplied path algorithms to define a new reference path that includes both ligand and pocket atoms. This updated path, consisting of 33 equidistant molecular configurations, provided a more comprehensive description for PCVs in SMD simulations of binding and unbinding events.

Before using this path in nonequilibrium simulations, we qualitatively evaluated the improvement given by the introduction of the pocket atoms in the path definition with Well-Tempered MetaDynamics simulations. From this analysis we understood that incorporating all binding pocket atoms, including side chains, into the reference path increased the system mobility on the free energy surface, facilitating easier ligand transitions between the bound and unbound states. This optimization led to more binding/unbinding events occurring within the same simulation time, accelerating convergence. In contrast, using only the ligand atoms in the path posed challenges, requiring significantly longer convergence times due to the extensive conformational rearrangements needed by the protein pocket atoms during binding/unbinding events. Well-Tempered MetaDynamics highlighted the complexity of defining optimal reference paths for intricate systems like the Abl-Gleevec complex. It also emphasized the necessity of including pocket atoms in the path definition for large, flexible systems.

Moreover, compared to previous kinase studies using Well-Tempered MetaDynamics and PCVs,[2] this study faced greater challenges. This reflected again the complexity of the Abl-Gleevec system, which is characterized by the remarkable conformational rearrangements

and intricacy of both the ligand and the receptor.

Then, following our protocol, we run SMD simulations along the refined reference path, which included both ligand and pocket atoms, for various simulation durations: (i) 10 ns, (ii) 50 ns, (iii) 100 ns, and (iv) 200 ns. A total of 50 binding and 50 unbinding simulations were performed, and statistical analysis was carried out to assess the convergence of the estimates based on the number of replicates.

The Jarzynski work profiles derived from the new reference path showed a significant reduction in dissipated work compared to those obtained in previous studies.[1] Figure S1 illustrates the Jarzynski work curves at 10 ns and 100 ns.

The new unbinding work curves resembled those from previous studies,[1] but with the inclusion of pocket atoms in the reference path, the final plateau was reached at lower Jarzynski work values, even for 10 ns SMD simulations (Figure S1 upper section). This suggested that incorporating the pocket led to a smoother opening of the binding pocket, even at higher pulling speeds. In contrast, binding simulations showed more significant differences: including the pocket in the reference path notably reduced dissipated work, especially for the longer 100 ns simulations. For these, total Jarzynski work values for binding were lower than for unbinding, with some values approaching zero (Figure S1 lower section).

FESs were obtained through the automated procedure at various pulling speeds, where $S(x)$ is normalized to the range $[0, 1]$ ($S(x) = 0$ corresponds to the bound state and $S(x) = 1$ to the unbound state). As expected, longer SMD simulations (100 and 200 ns) resulted in FESs with a plateau at lower free energy values, corresponding to the fully solvated ligand state, due to lower dissipated work at slower pulling speeds.

For completeness of results, FESs were also computed using the monodirectional Jarzynski estimator for both events. The JE-derived FESs from slower pulling speeds (100 and 200 ns) reached the solvated state at lower free energy values, but these plateaus occurred at higher values compared to the CFT FESs (Figure S2), due to higher dissipated work dur-

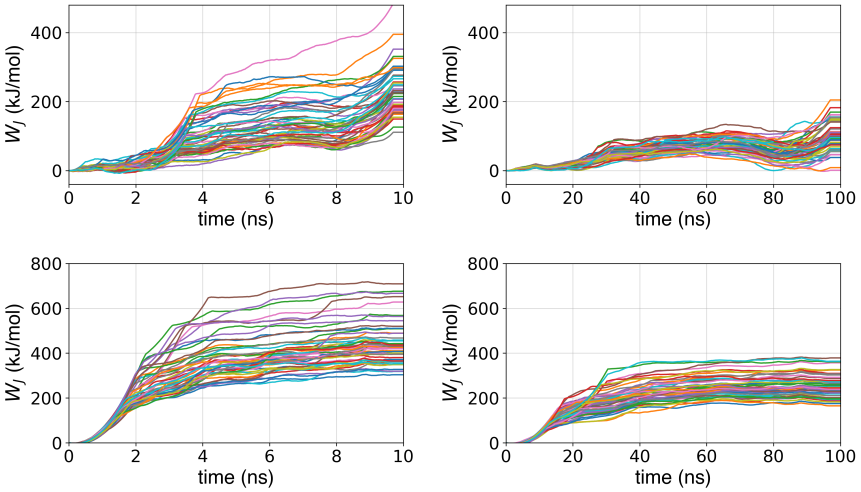

Figure S1: Jarzynski work profiles of Abl-Gleevec for binding (upper section) and unbinding simulations (lower section), measured over simulation times of 10 and 100 ns.

ing unbinding. For binding simulations, at 10 ns, the high pulling speed caused significant dissipated work, leading to inaccurate FESs and an inverted profile compared to unbinding. Slower pulling speeds (100 and 200 ns) reduced dissipation, bringing cumulative Jarzynski work values closer to zero and resulting in a more accurate FES. However, even at 100 ns, considerable dissipated work remained, necessitating 200 ns simulations to achieve profiles comparable to unbinding results.

The binding free energies derived from the FESs (after volume correction) are summarized in Table S1. The CFT estimator effectively mitigates the high dissipated work seen during both binding and unbinding transformations, providing more reliable estimates across all simulation times. However, despite applying the CFT estimator to the longer 200 ns SMD simulations, the resulting binding free energy of -21 $\pm$ 3 kcal/mol still deviates from the experimental value.

Although incorporating pocket atoms into the reference path used with PCVs during SMD simulations significantly reduces dissipated work, the resulting binding free energy estimates, even for longer simulations, are still less-than-ideal. While including pocket atoms is essential for systems with large, flexible binding pockets, it alone is insufficient to define the optimal

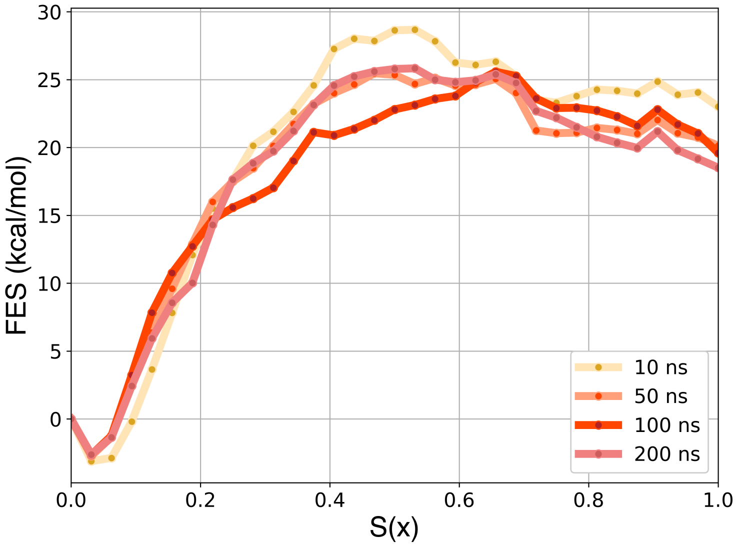

Figure S2: Free energy profiles along $S(x)$ obtained by applying CFT to SMD of different time length with PCVs for Abl-Gleevec.

Table S1: Standard binding free energies (kcal/mol) for Abl-Gleevec estimated using JE and CFT for increasing simulation times.

	**10 ns**	**50 ns**	**100 ns**	**200 ns**
**JE binding**	17 ± 10	8 ± 8	0 ± 10	-4 ± 6
**JE unbinding**	-66 ± 2	-48 ± 1	-43.1 ± 0.8	-39 ± 1
**CFT**	-26 ± 5	-23 ± 4	-23 ± 5	-21 ± 3

minimum free energy path. For complex systems with challenging conformational dynamics, defining the reference path becomes crucial to the accuracy and convergence of binding free energy estimates. Thus, further refinements are needed in the pipeline to achieve optimal path definitions for large systems.

Work Profiles and FESs

Following the optimal path definition rules, we obtained a refined path composed of 42 molecular configurations. Figure S3 shows the Jarzynski work profiles of Abl-Gleevec for binding (upper section) and unbinding simulations (lower section), measured over simulation times of 100 and 200 ns.

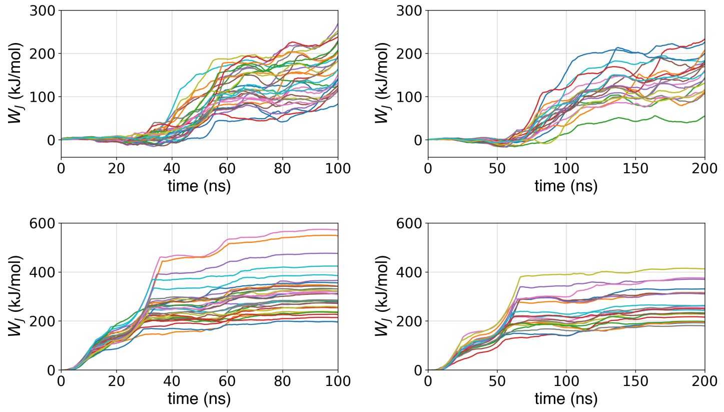

Figure S3: Jarzynski work profiles of Abl-Gleevec for binding (upper section) and unbinding simulations (lower section), measured over simulation times of 100 and 200 ns

The CFT Free energy profiles along $S(x)$, along with their corresponding bootstrap error estimates, are presented in Figure S4

DFG-flip contribution

During the 200 ns simulations, conformational changes were observed in both the P-loop and A-loop, whereas the DFG motif exhibited greater stability throughout the simulations. These fluctuations are illustrated in Figure S5, where the DFG sequence is highlighted in

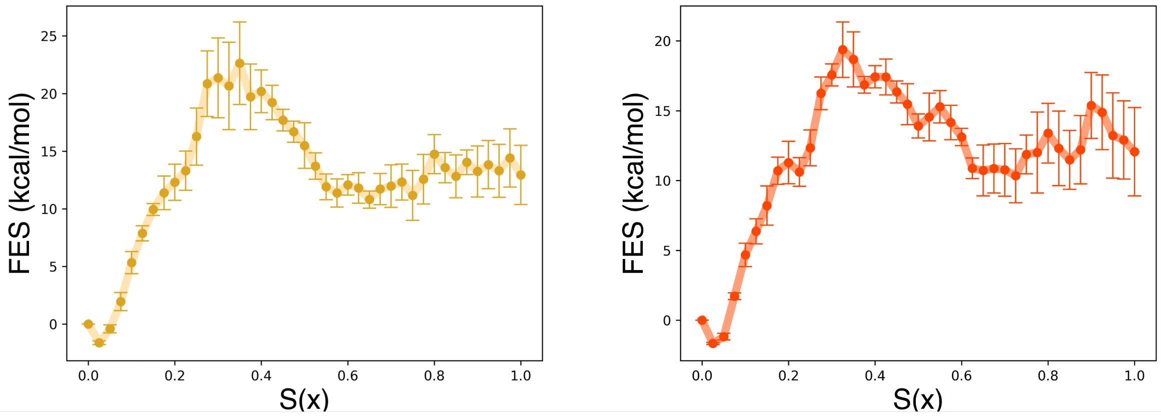

Figure S4: Free energy profiles along $S(x)$ obtained by applying CFT to SMD of 100 and 200 ns with the refined PCVs for Abl-Gleevec, along with their corresponding bootstrap errors.

red. Root-mean-square fluctuations (RMSF) analysis confirms the dynamic behavior of the loops compared to the stability of the DFG motif (out conformation).

The contribution of the DFG-flip had to be accounted for in the calculation of the standard binding free energy. Specifically, the standard binding free energy of the Abl-Gleevec complex $\Delta F^{\circ}_{bind,in\rightarrow out}$ corresponds to the following reaction:

$$Abl_{in} + Gleevec \rightarrow Abl_{out} : Gleevec \tag{1}$$

However, we computed $\Delta F^{\circ}_{bind,out}$ corresponding to a reaction where the DFG-motif of Abl is in the out conformation in both the bound and unbound states:

$$Abl_{out} + Gleevec \rightarrow Abl_{out} : Gleevec \tag{2}$$

Therefore, by adding $\Delta F_{in\rightarrow out}$ to $\Delta F^{\circ}_{bind,out}$, we can recover the standard binding free energy accounting for the DFG-flip:

$$\Delta F^{\circ}_{bind,in\rightarrow out} = \Delta F^{\circ}_{bind,out} + \Delta F_{in\rightarrow out} \tag{3}$$

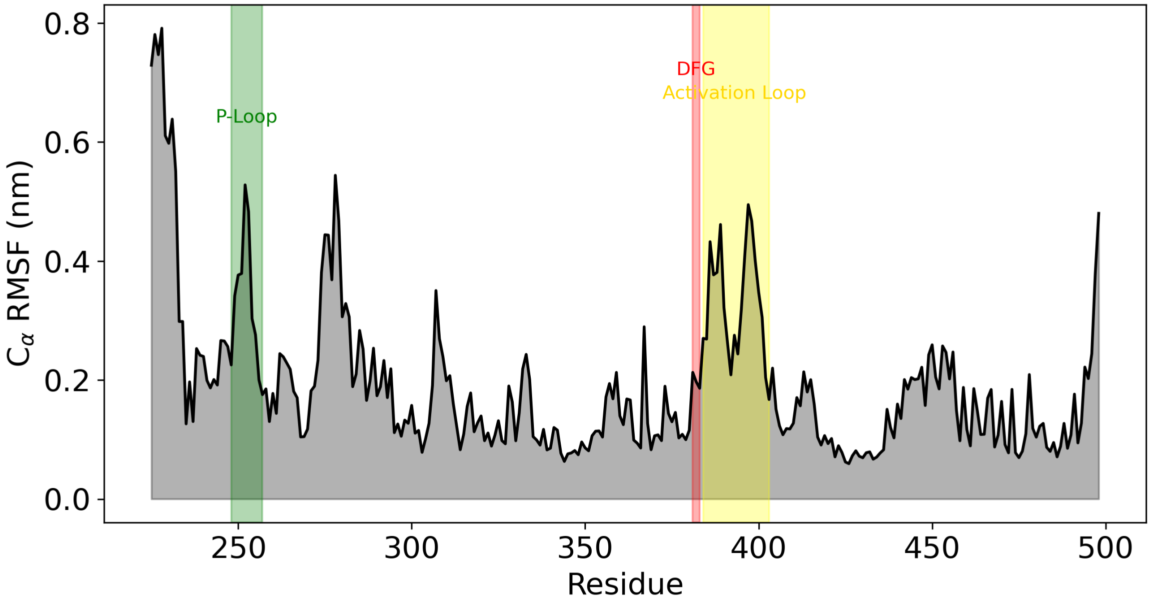

Figure S5: RMSF of Abl. Fluctuations of the P-loop (green) and of the A-loop (yellow), with highlighted in red the DFG sequence. Conformational rearrangements occur for the loops during simulations, while the DFG sequence remains more stable in its out conformation

Additional results for RNA-ligand systems

In this section, additional information and results of RNA-ligand systems are reported. Particularly: the Debye-Hückel interaction energy profiles, the Root Mean Square Fluctuation analysis for cognate and synthetic ligands, the selected atoms for the coursed grain like unbinding path, the 3D structure of Riboswitch preQ1 with its cognate ligand, and the work profiles obtained from SMD simulations. Moreover, for completeness of the results, the FESs obtained from mono-directional Jarzynski estimator are also presented.

Debye-Hückel interaction energy profile

Figure S6 reports the Debye-Hückel interaction energy during the ABMD simulations. These plots demonstrate the reduction of the interaction energy as the ligands unbind, with final values of 1.73 and 1.14 kcal/mol for the cognate and synthetic ligands, respectively.

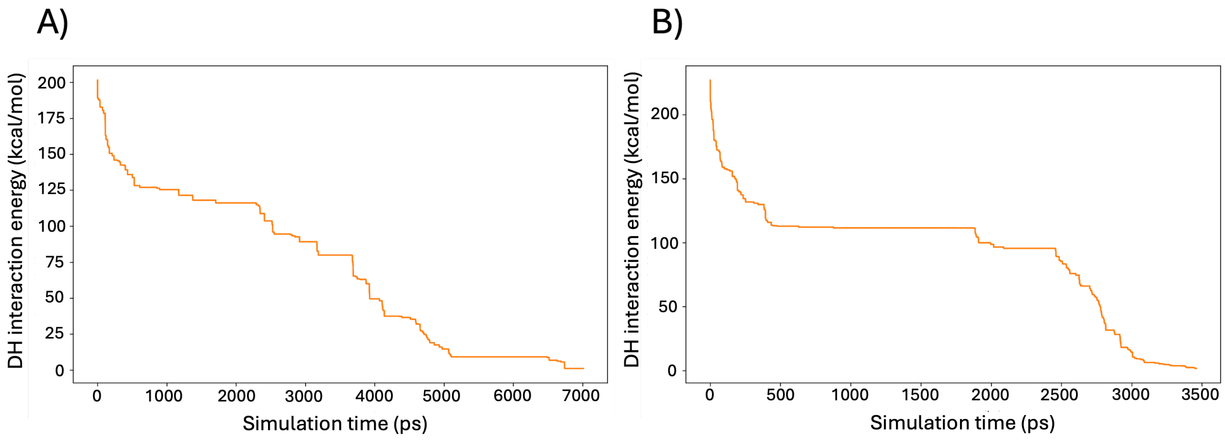

Figure S6: Debye-Hückel interaction energy profile of the cognate (A) and synthetic (B) ligands over simulation time

Analysis of PCV and Path definition

To identify appropriate atoms for alignment, we conducted three unbiased 100 ns MD simulations and combined the data to compute residue-specific root-mean-square fluctuations.

Residues with RMSF values below a threshold of 1.8 Å were selected (Figure S7), and a subset of their atoms was chosen for alignment. The selected atoms for the coarse-grain-like unbinding path are illustrated in Figure S8. Specifically, we aligned the RNA backbone using the P and $C1'$ carbon atoms. Importantly, this approach aligned conceptually with the selection criteria applied in the protein system.

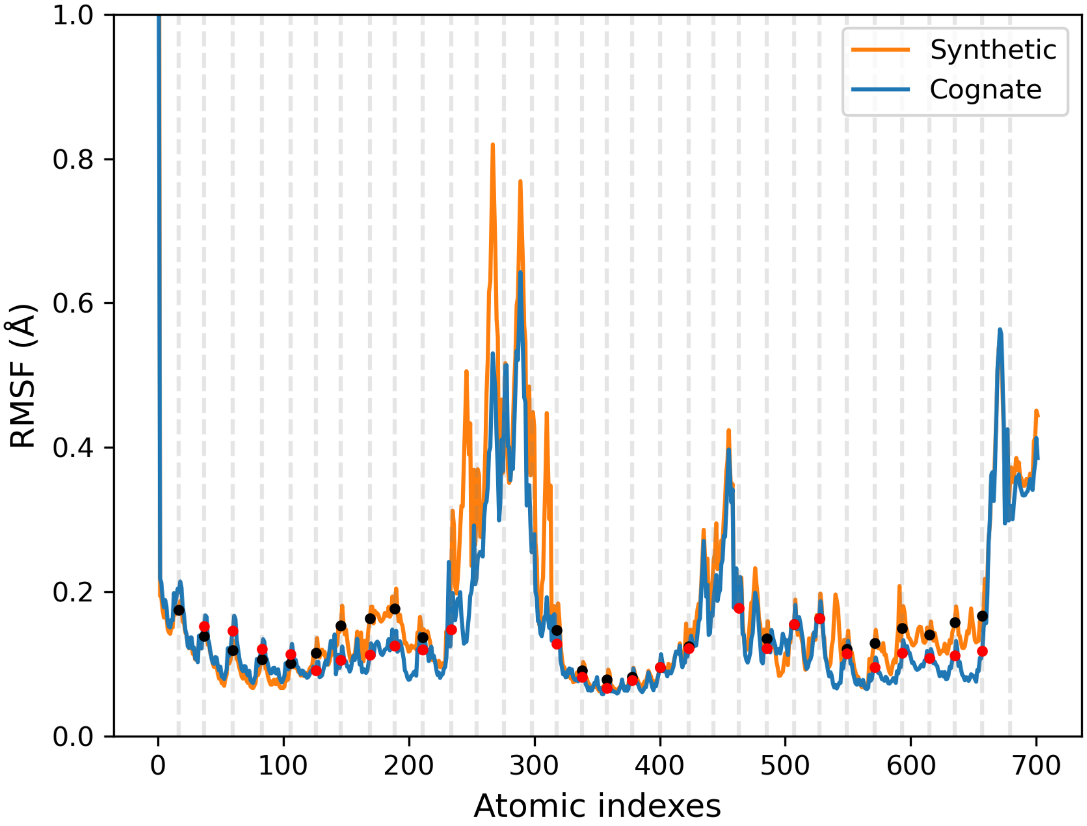

Figure S7: Root Mean Square Fluctuation analysis for cognate (blue) and synthetic (orange) ligands calculated across three combined 100 ns unbiased trajectories. Black and red dots represent phosphate atoms of residues with RMSF $<$ 0.18 Å, included in the unbinding pathway.

3D structure of Riboswitch

The 3D structure of the riboswitch is depicted in Figure S9, emphasizing its key structural elements, specifically:

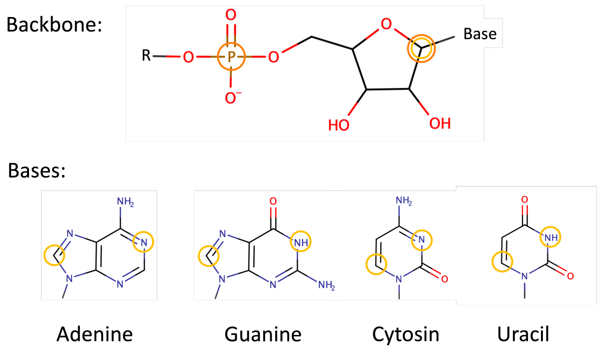

Figure S8: Schematic representation of the selected atoms for the coursed grain like unbinding path. It is represented in orange the atom selection for the alignment and in yellow the ones for the RMSD calculation

- Loop 1: residues U6-U7-C8;
- Loop 2: residues U12-U13-A14-U15-A16-C17;
- Loop 3: residues A23-U24-A25-A26-A27-A28-A29-A30;
- Stem 1: residues A1-G2-A3-G4-G5 and C18-C19-U20-C21-U22;
- Stem 2: residues U9-A10-G11 and C31-U32-A33-A34.

.

Residues are colored according to their secondary structure.

Moreover, figure S10 depicts the 2D representation of hydrogen bonds formed by the synthetic ligand within the riboswitch-preQ1 complex.

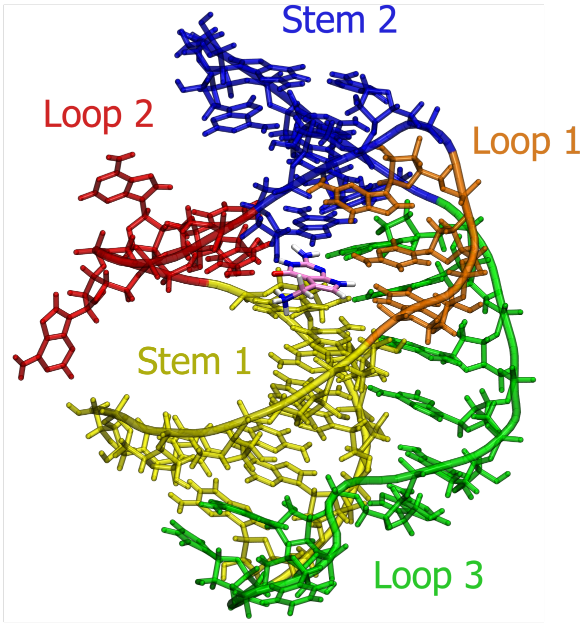

Figure S9: 3D structure of Riboswitch preQ1 with its cognate ligand (Q1) with residues colored according to their secondary structure.

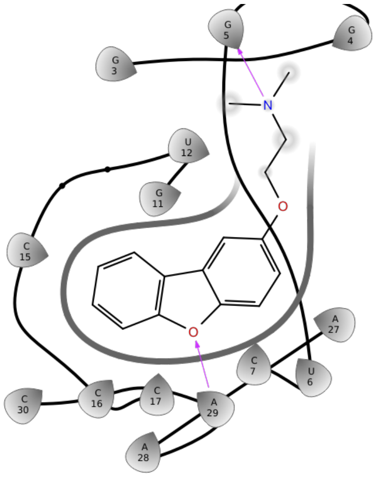

Figure S10: 2D representation of the hydrogen bonds established by the synthetic ligand within the riboswitch-preQ1 complex.

Work Profiles and FESs

As typically observed, inspection of the work profiles revealed that work in the unbinding simulations increases almost monotonically (Figure S11). This indicates the presence of an energy barrier that needs to be overcome by the ligand to disrupt the interactions of

the bound state and escape the binding pocket. Once the ligand has reached the unbound state and is fully solvated, the work profile reaches a plateau at around 45 ns. In contrast, at the beginning of the binding simulations, the ligand is in the solvent, corresponding to almost null work. When the ligand approaches the RNA target (simulation time 45 ns), the work increases until the bound state is reached. Different replicas exhibit different work dissipation, reflecting the complexity of the ligand association process. However, the increase in the work to reach the binding state is minimal, indicating minor difficulties for the ligand to adopt the binding mode.

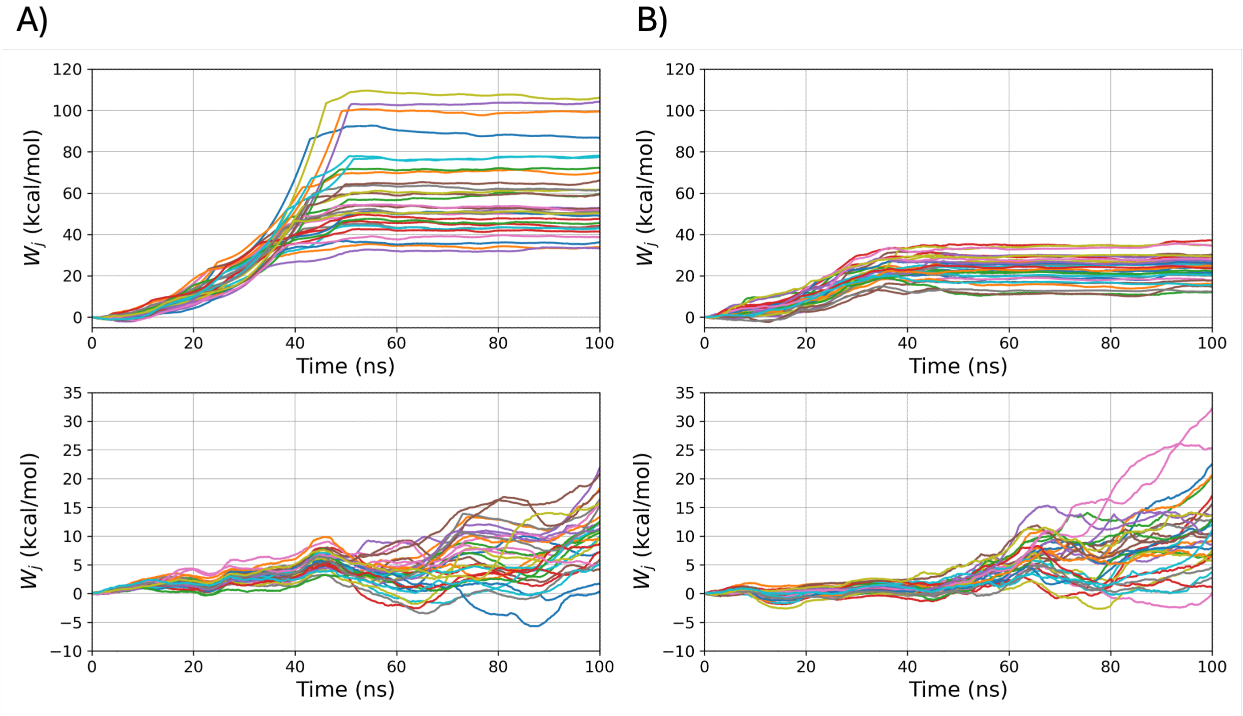

Figure S11: Jarzynski work profiles for the cognate ligand (A) and synthetic ligand (B) regarding unbinding (upper section) and binding (lower section) simulations measured over a simulation time of 100 ns in TIP4P-D.

Furthermore, the work profiles obtained using the TIP3P water model (as a replacement for the TIP4P-D water model) are presented in Figure S12.

FESs were obtained through the automated procedure and are reported in Figures S13 and Figure S14

Finally, Jarzynski work profiles and Free energy profiles for the tautomer of the cognate

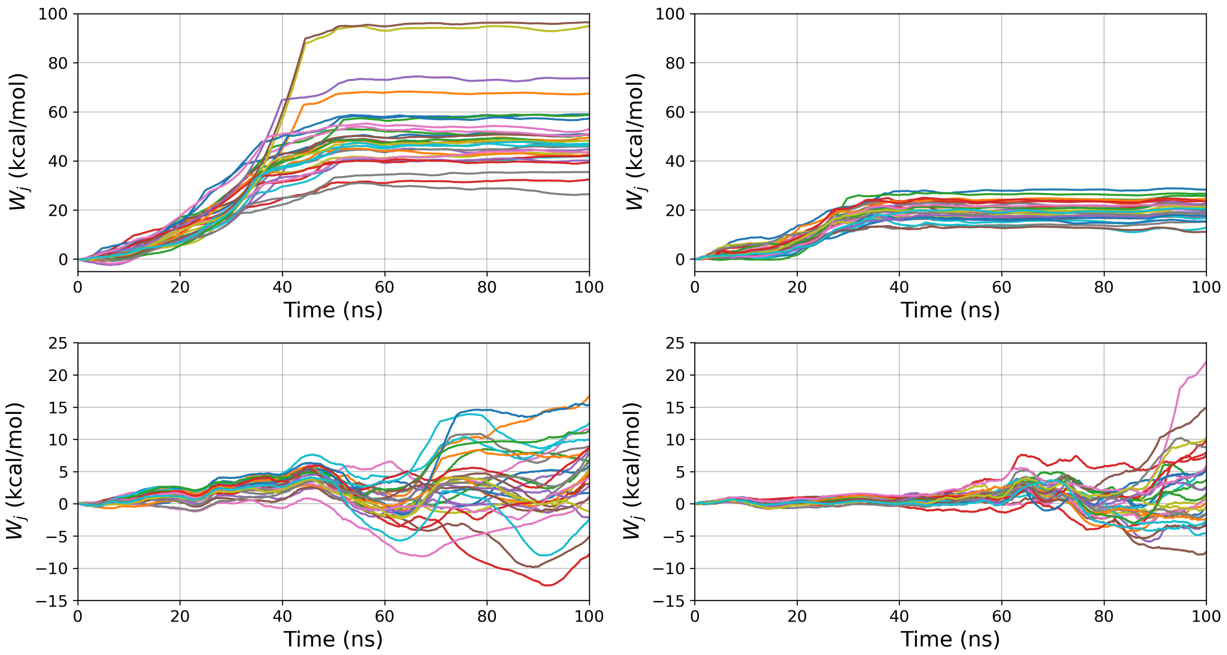

Figure S12: Jarzynski work profiles for the cognate ligand (A) and synthetic ligand (B) regarding unbinding (upper section) and binding (lower section) simulations measured over a simulation time of 100 ns in TIP3P.

ligand are reported in Figure S15 and Figure S16, respectively.

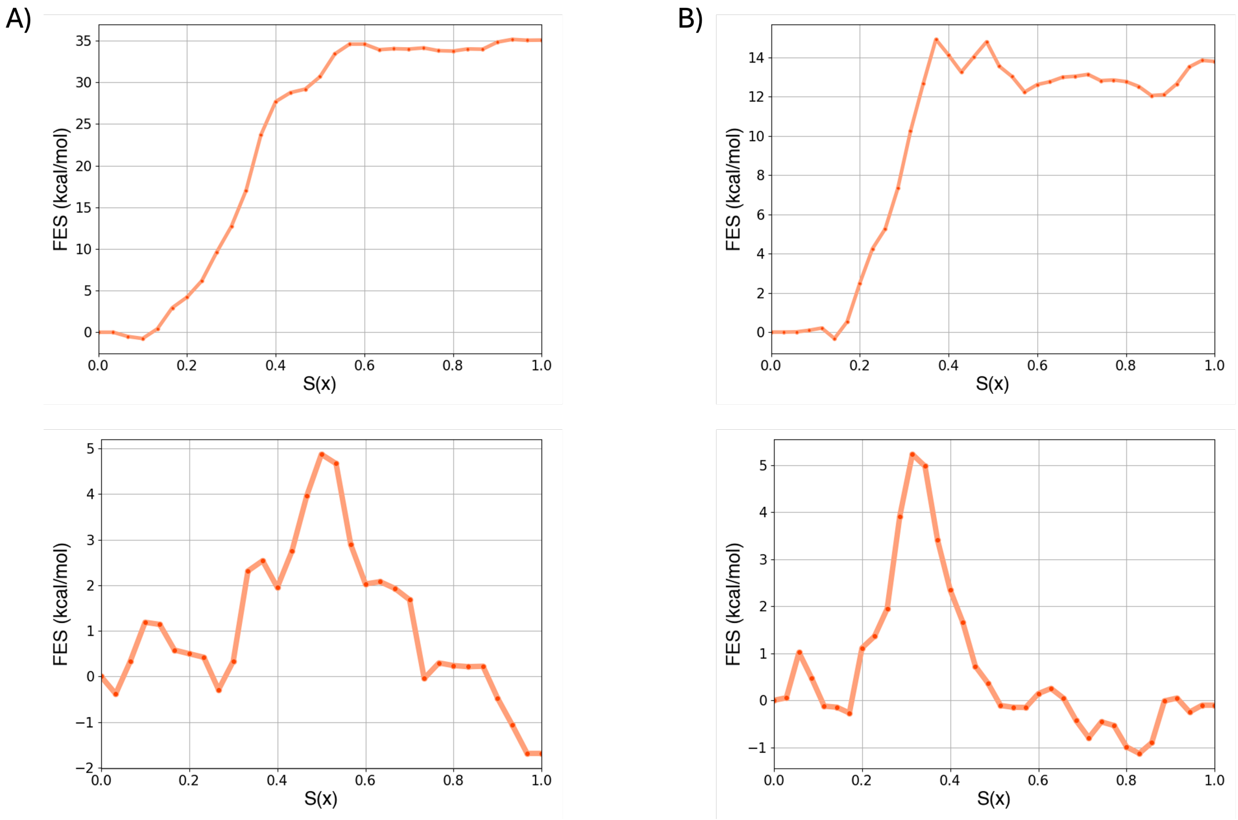

Figure S13: FES reconstruction using Jarzynski Equality (JE) for both unbinding (upper section) and binding (lower section) simulations in TIP4P-D water model for the cognate (A) and synthetic (B) ligands.

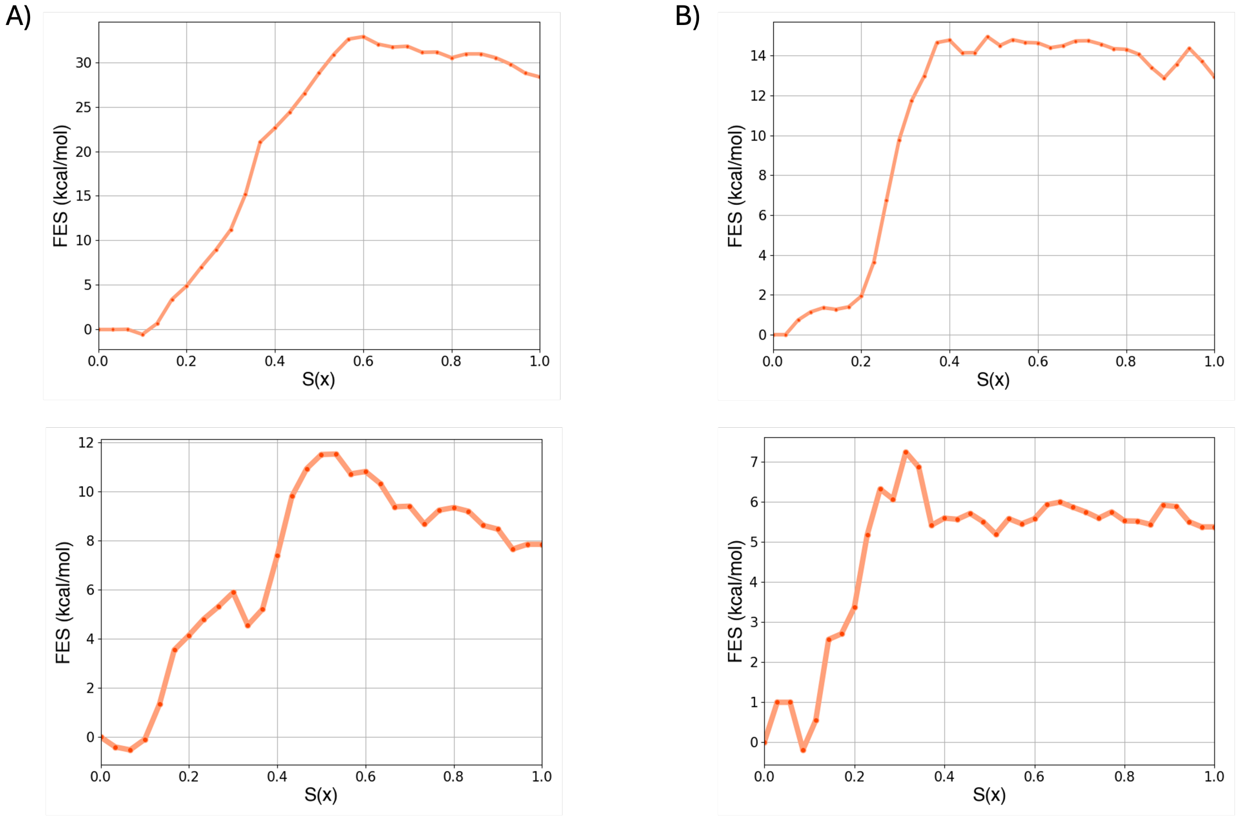

Figure S14: FES reconstruction using Jarzynski Equality (JE) for both unbinding (upper section) and binding (lower section) simulations in TIP3P water model for the cognate (A) and synthetic (B) ligands.

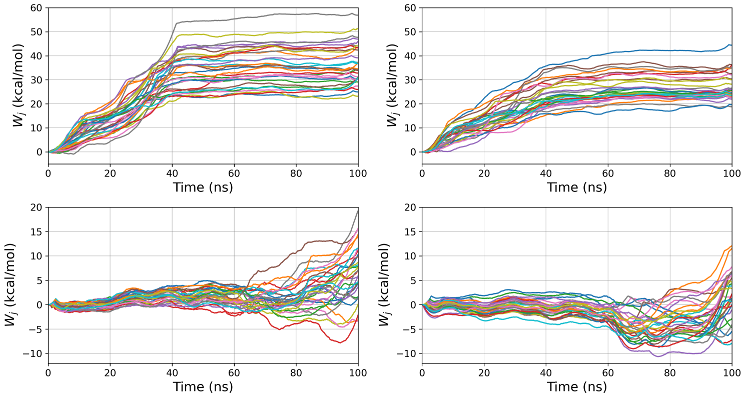

Figure S15: Jarzynski work profiles for the tautomer of the cognate ligand regarding unbinding (upper section) and binding (lower section) simulations measured over a simulation time of 100 ns in TIP4P-D (A) and TIP3P (B).

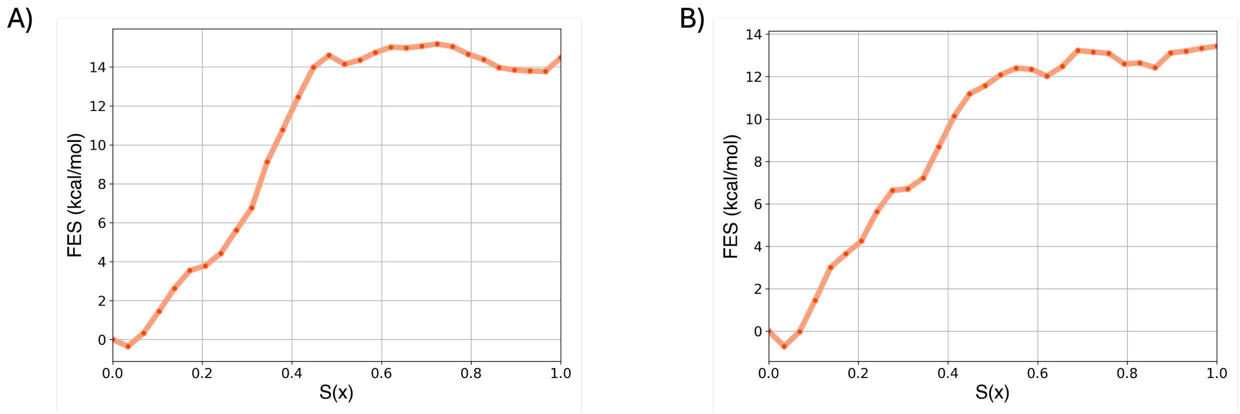

Figure S16: Free energy profiles along S(x) obtained by applying CFT to SMD simulations in TIP4P-D (A) and TIP3P (B) for the tautomer of the cognate ligand.

Well-Tempered MetaDynamics additional results

To further check our findings, we also performed Well-Tempered MetaDynamics simulations with PCVs using the same reference pathways employed in SMD simulations. Unlike nonequilibrium methods relying on Crooks/Jarzynski estimators, which are dependent on work values, Well-Tempered MetaDynamics employs a time-varying Hamiltonian without this dependency, offering an interesting point of comparison. After fine-tuning the Well-Tempered MetaDynamics parameters, convergence was achieved and FESs were successfully reconstructed.

Well-Tempered MetaDynamics of Abl-Gleevec

In Figure S17, the time series of $S(x)$ along the metadynamics trajectories for Abl-Gleevec system is reported, while in Figure S18 the free energy profile as function of $S(x)$ is represented. These plots demonstrate that Gleevec is able to move away from the protein, allowing an exhaustive sampling of the unbound state. This qualitative observation underscores the importance of the newly introduced criteria in the reference path definition.

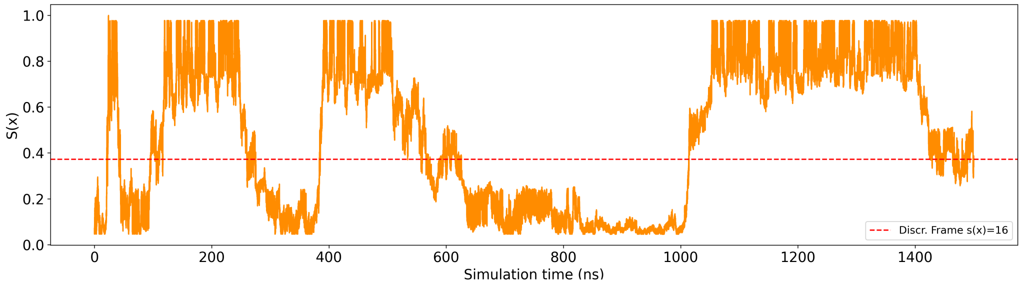

Figure S17: Time series of the collective variable S(x) along the metadynamics trajectories for Abl-Gleevec system. The red dotted line represents the discriminating frame used for free energy calculations

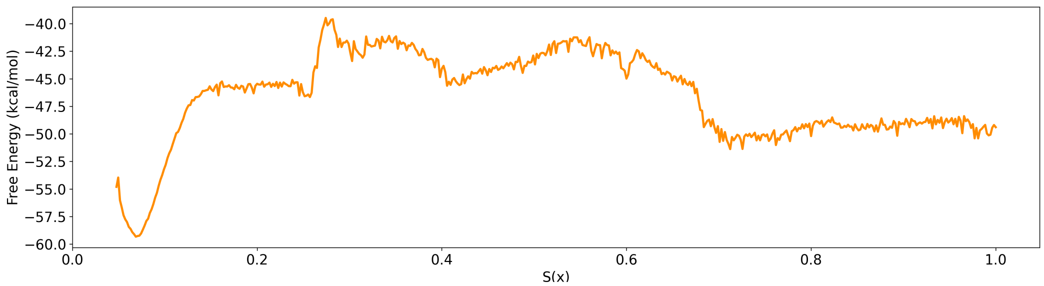

Figure S18: Reweighted free energy profile as a function of the collective variable S(x) from metadynamics simulation for Abl-Gleevec system.

Well-Tempered MetaDynamics of RNA-ligands

Figure S19 shows the time series of $S(x)$ along the metadynamics trajectories of the cognate (top) and synthetic (bottom) ligands. The red dotted line indicates the discriminating frame used for the free energy calculations. Moreover, Figure S20 illustrates the free energy profile as a function of $S(x)$ for the cognate ligand (top) and the synthetic ligand (bottom).

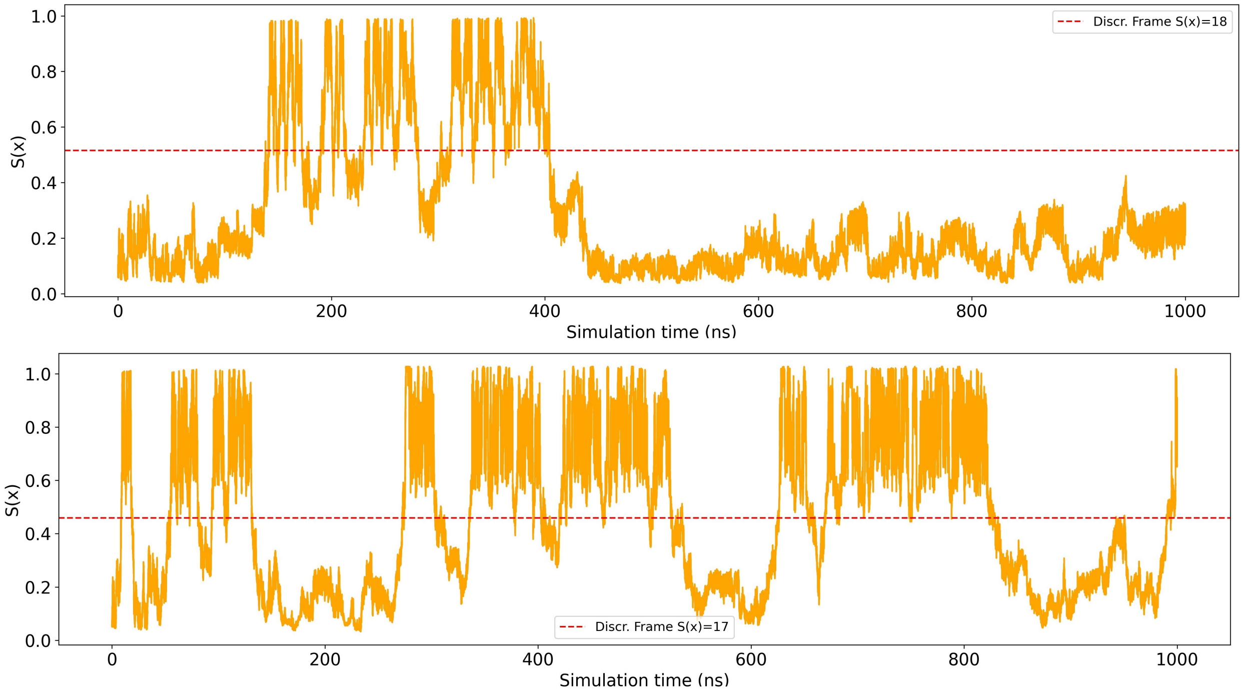

Figure S19: Time series of the collective variable S(x) along the metadynamics trajectories for the cognate (top) and synthetic (bottom) ligands. The red dotted line represents the discriminating frame used for free energy calculations in both cases.

For the tautomer, Figure S21 displays the time series of the collective variable $S(x)$ along

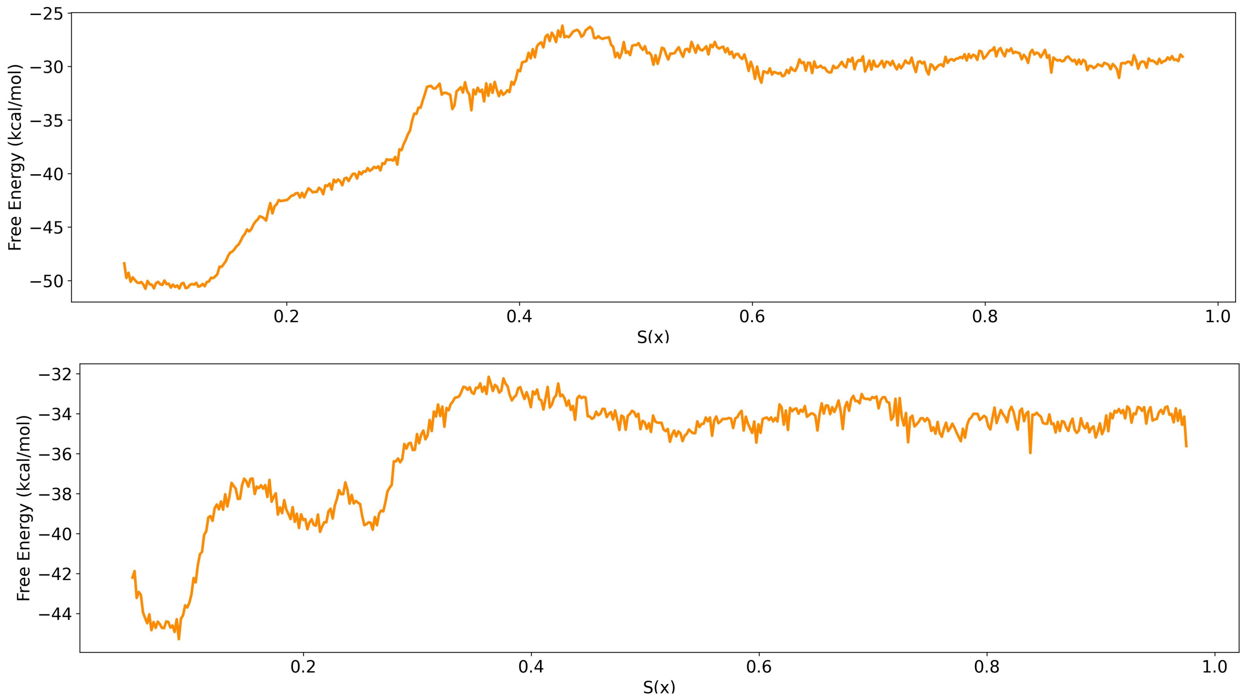

Figure S20: Reweighted free energy profile as a function of the collective variable S(x) from metadynamics simulation for the cognate (top) and synthetic (bottom) ligands.

the metadynamics trajectory, while Figure S22 presents the reweighted free energy profile as a function of $S(x)$.

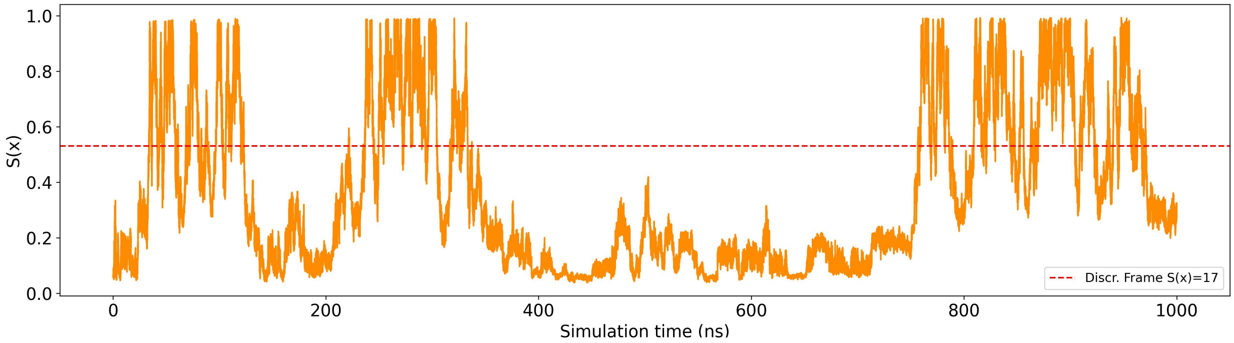

Figure S21: Time series of the collective variable S(x) along the metadynamics trajectory for the tautomer. The red dotted line indicates the discriminating frame used for free energy calculations.

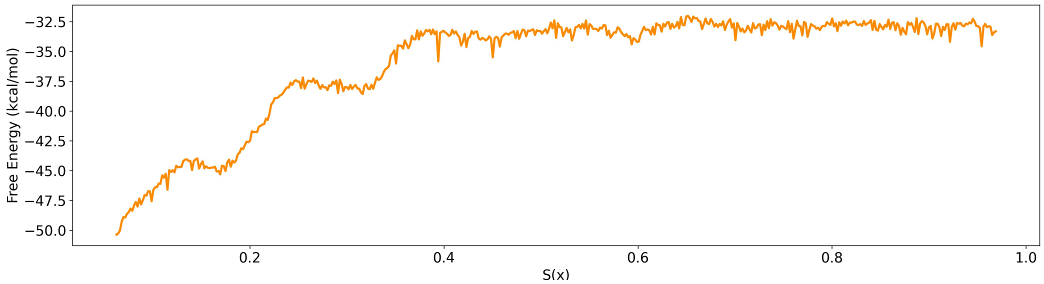

Figure S22: Reweighted free energy profile as a function of the collective variable S(x) from Well-Tempered MetaDynamics simulation for the tautomer.

Table S2: Standard binding free energies (kcal/mol) for the cognate and synthetic ligands using the TIP4P-D water model in MetaD simulations and TIP4P-D and TIP3P in Steered Molecular Dynamics.

	**SMD (TIP4P-D)**	**SMD (TIP3P)**	**MetaD**
**Cognate**	-17.2 ± 1	-18.6 ± 3	-19.4 ± 2
**Synthetic**	-5.6 ± 1	-8.7 ± 0.7	-7.4 ± 0.9

Moreover, to compare the results reported in S2, the two-means Z-test results are reported in Table S3.

Table S3: Two-means Z-test results

		**Z value**
cognate	SMD TIP3P - MetaD	1.5
cognate	SMD TIP4P-D - MetaD	3.0
synthetic	SMD TIP3P - MetaD	1.6
synthetic	SMD TIP4P-D - MetaD	4.3

Possible sources of errors for the cognate ligand - additional results

Figure S23 displays the atomic charge differences between RESP charges (calculated using PlayMolecule) and AM1-BCC charges (calculated with Antechamber) for the cognate ligand.

Figure S24 illustrates the FES reconstruction using Jarzynski estimator for both unbinding (upper section) and binding (lower section) simulations, comparing TIP4P-D (A) and TIP3P (B) water models for the cognate ligand's tautomer.

Figure S25 presents the FES reconstruction using Jarzynski estimator for unbinding (upper section) and binding (lower section) simulations, comparing TIP4P-D (A) and TIP3P (B) water models for the tautomer of the cognate ligand.

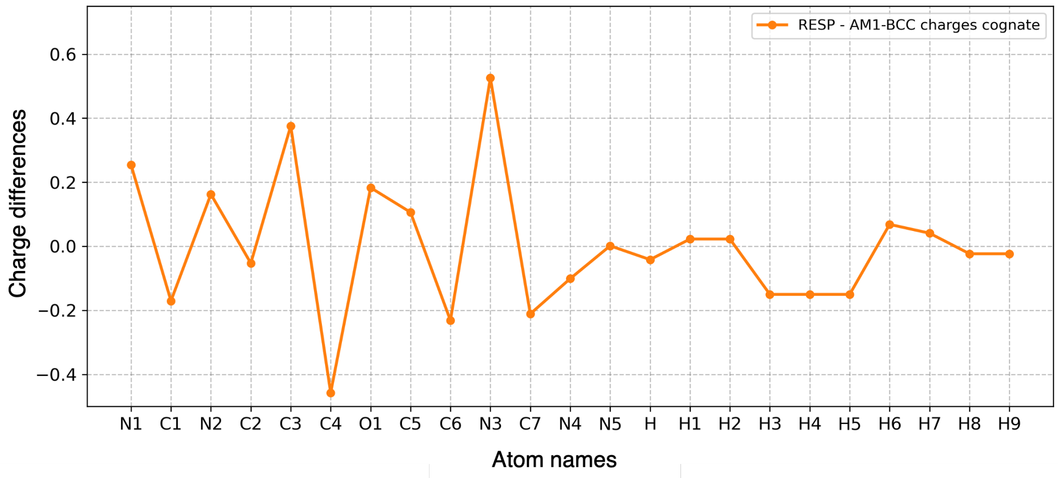

Figure S23: Atomic charge differences between RESP charges (calculated by PlayMolecule) and AM1-BCC charges (calculated by antechamber) for the cognate ligand.

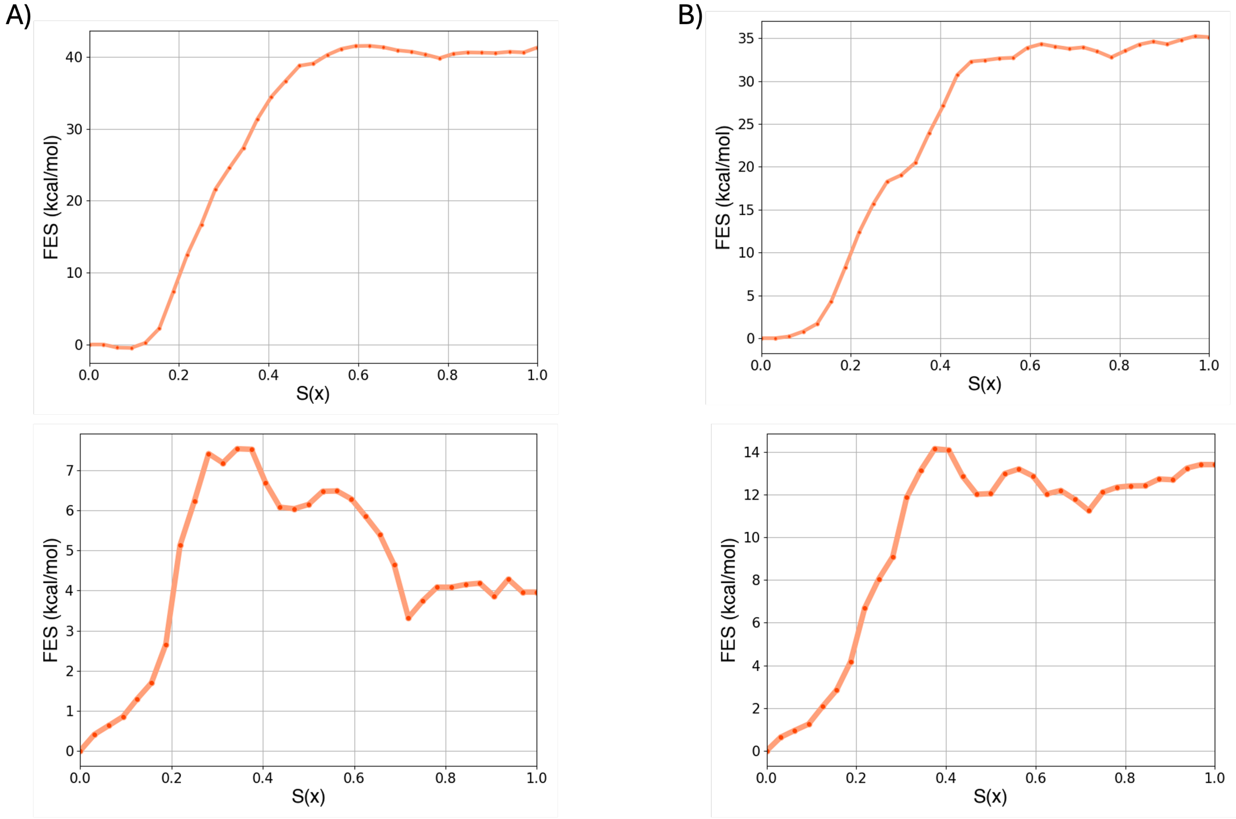

Figure S24: FES reconstruction using Jarzynski Equality (JE) for both unbinding (upper section) and binding (lower section) simulations in TIP4P-D (A) and TIP3P (B) water model for the cognate ligand with RESP charges

Additional parameters

To ensure comprehensiveness and consistency of our results and definitively exclude the parameterization procedure (i.e. the one in Ref.[3] and the one in the present work) as a source of error, we reparametrized all ligands, i.e the synthetic and the cognate one (the latter in both tautomeric forms N1 and N2). Thus, instead of relying on the topologies from Ref.,[3] we reconstructed the ligand topologies from scratch using the AM1-BCC charge model and GAFF. The only appreciable differences were in ligand partial charges, with slight differences compared to those in Ref.[3] Additional simulations were performed for all complexes using this newly generated set of parameters. Simulations were conducted in both TIP4P-D and TIP3P water models. With these updated parameters, we repeated the entire workflow, including ABMD, path definition, two-sided SMD simulations, and final binding

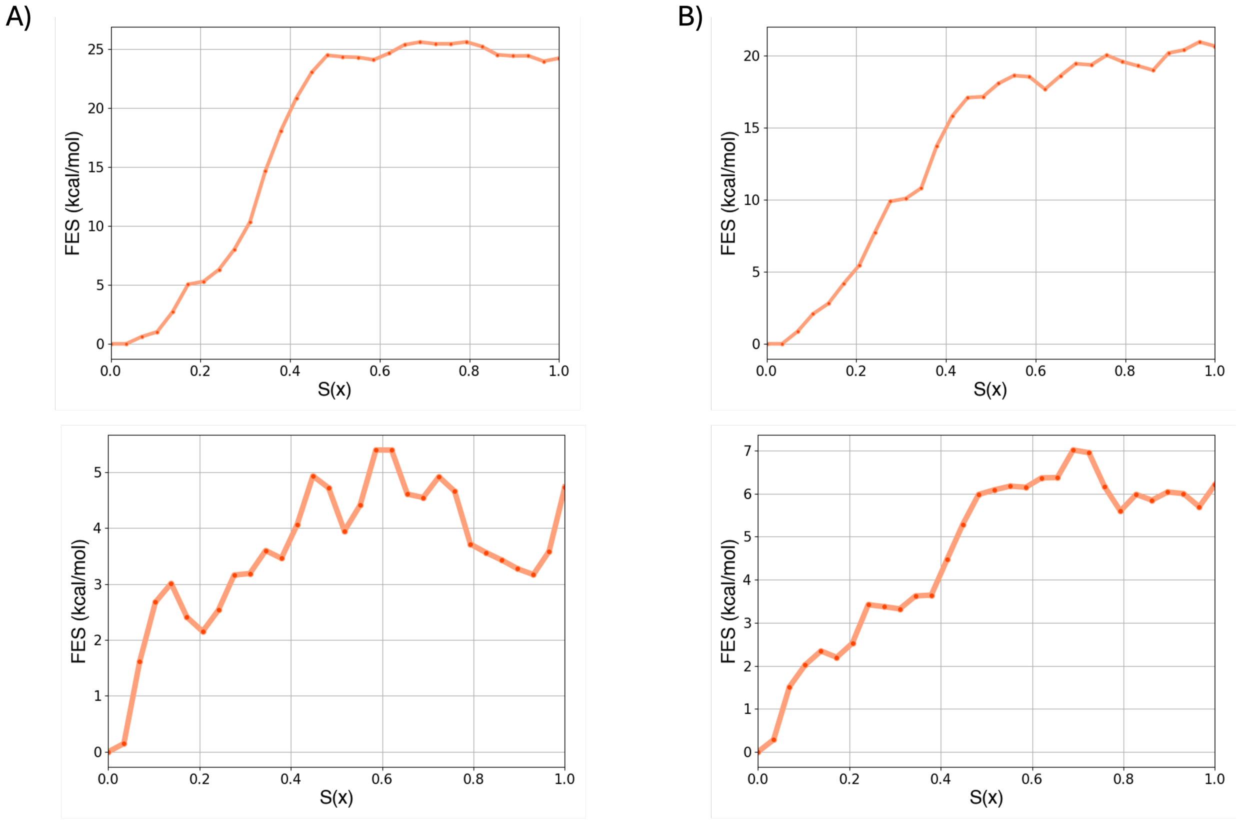

Figure S25: FES reconstruction using Jarzynski Equality (JE) for both unbinding (upper section) and binding (lower section) simulations in TIP4P-D (A) and TIP3P (B) water model for the tautomer of the cognate ligand.

free energy estimation. The results from these additional simulations are summarized in Figure S26. The consistency of these results with previous findings provides further evidence that parameter selection is not the primary source of error for the cognate ligand. Moreover, it demonstrates that our nonequilibrium pipeline is both reproducible and stable.

Moreover, we employed Jaguar, a Schrödinger program, to study the two tautomeric forms of the cognate ligand, N1 and N2. Specifically, the hydrogen atom can be positioned on either of the pyrimidine ring's nitrogen atoms through tautomerization. To evaluate the coexistence of these two tautomers, we performed ab initio quantum mechanical calculations using Jaguar.[4] The B3LYP-3D method, along with the 6-311G++** QM basis set, was applied. Moreover, the Poisson Boltzmann Finite element method (PBF) was used to model solvation effects. The difference in hydration free energy between the QM-optimized N1 and

N2 tautomer is 1.85 kcal/mol, in favor of N1.

Table S4: Binding free energies (kcal/mol) for the cognate ligand in the tautomeric form N1 with RESP and AM1-BCC charges and the tautomeric form N2 with AM1-BCC charges in different water models.

	**TIP4P-D**	**TIP3P**
**N1 - RESP**	-21.2 ± 1	-21.4 ± 1
**N1 -AM1-BCC**	-17.2 ± 1	-18.6 ± 3
**N2 -AM1-BCC**	-13.2 ± 1	-11.9 ± 1

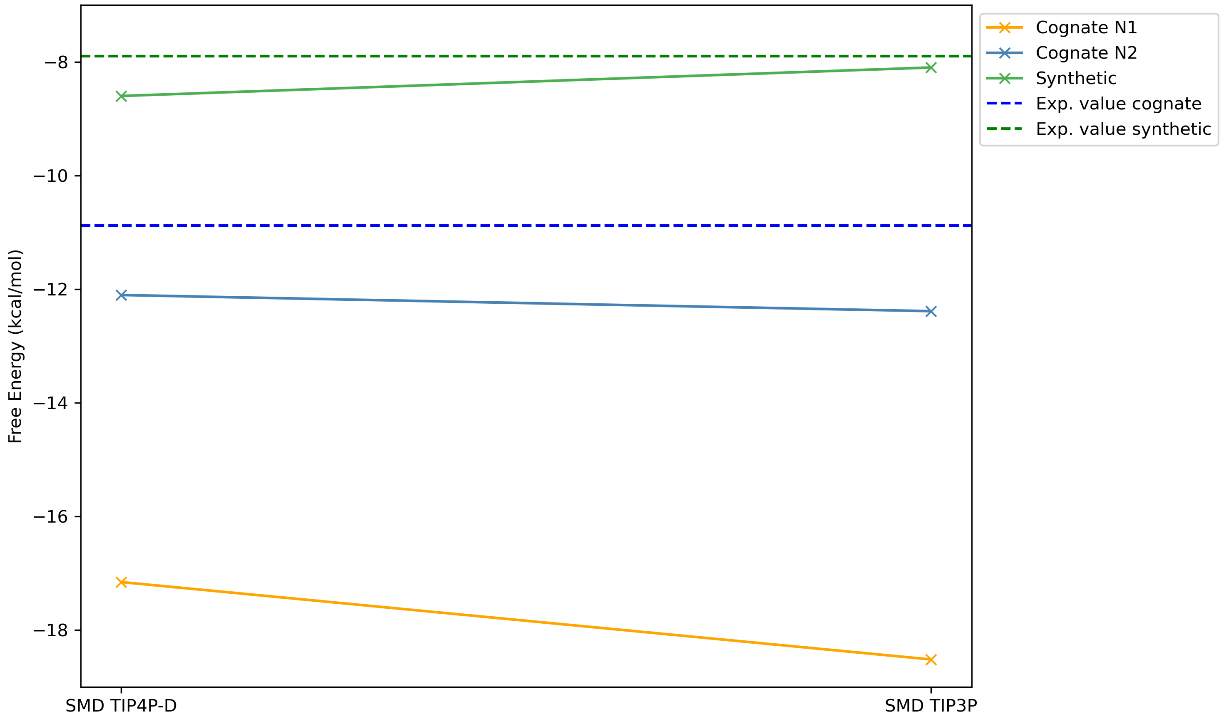

Figure S26: Binding free energies results. Dashed lines indicate experimental values (green: synthetic, blue: cognate). The x-axis represents the calculation method (SMD in TIP4P-D and TIP3P), while the y-axis shows the corresponding binding free energy. Ligands are color-coded: synthetic (green), cognate with AM1-BCC charges (orange) and cognate tautomer (light blue).

Hydrogen bonds analysis

Analysis of 500 ns Plain MD simulations for the two tautomers, N1 and N2, reveals distinct hydrogen bonding patterns within the binding pocket over time. Figures S27 and S28 show

these patterns for the two forms. Specifically, Figure S27 (N1 tautomer) shows that the number of hydrogen bonds remained stable at approximately seven throughout the simulation time. In contrast, Figure S28 (N2 tautomer) demonstrates that the average number of hydrogen bonds was five.

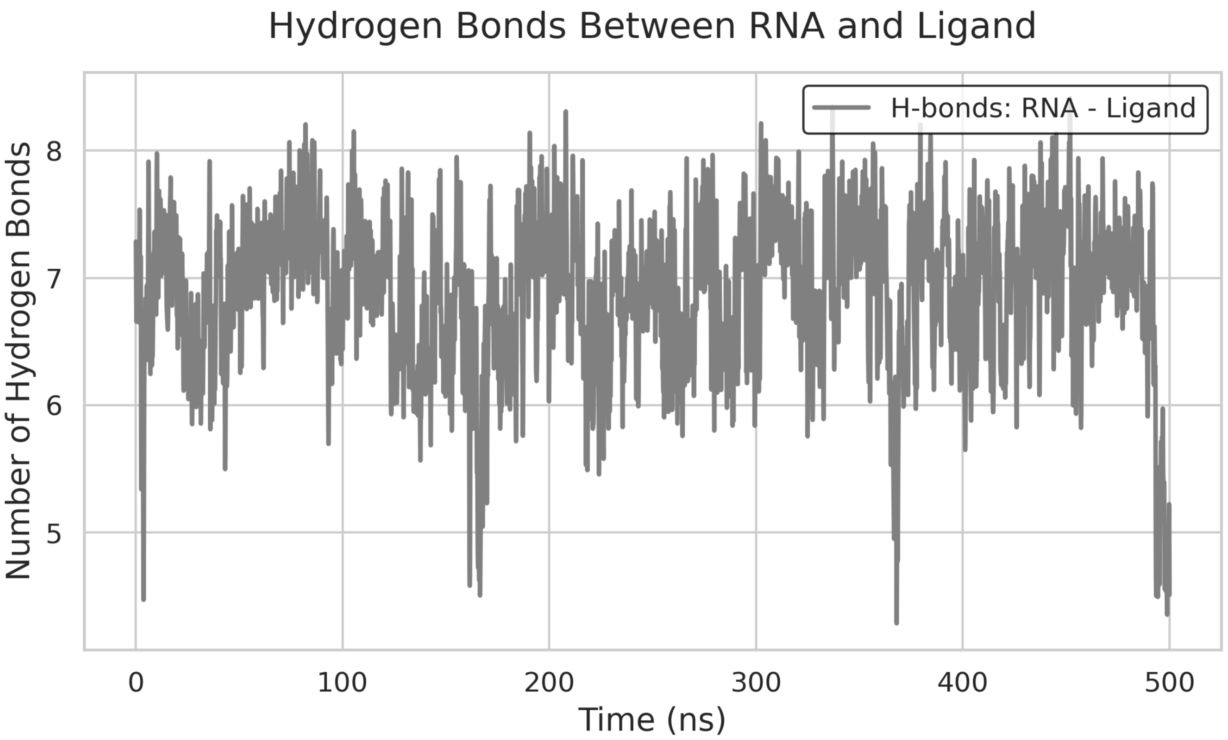

Figure S27: The number of hydrogen bonds for the N1 tautomer remained consistently around seven throughout the 500 ns of plain MD simulations.

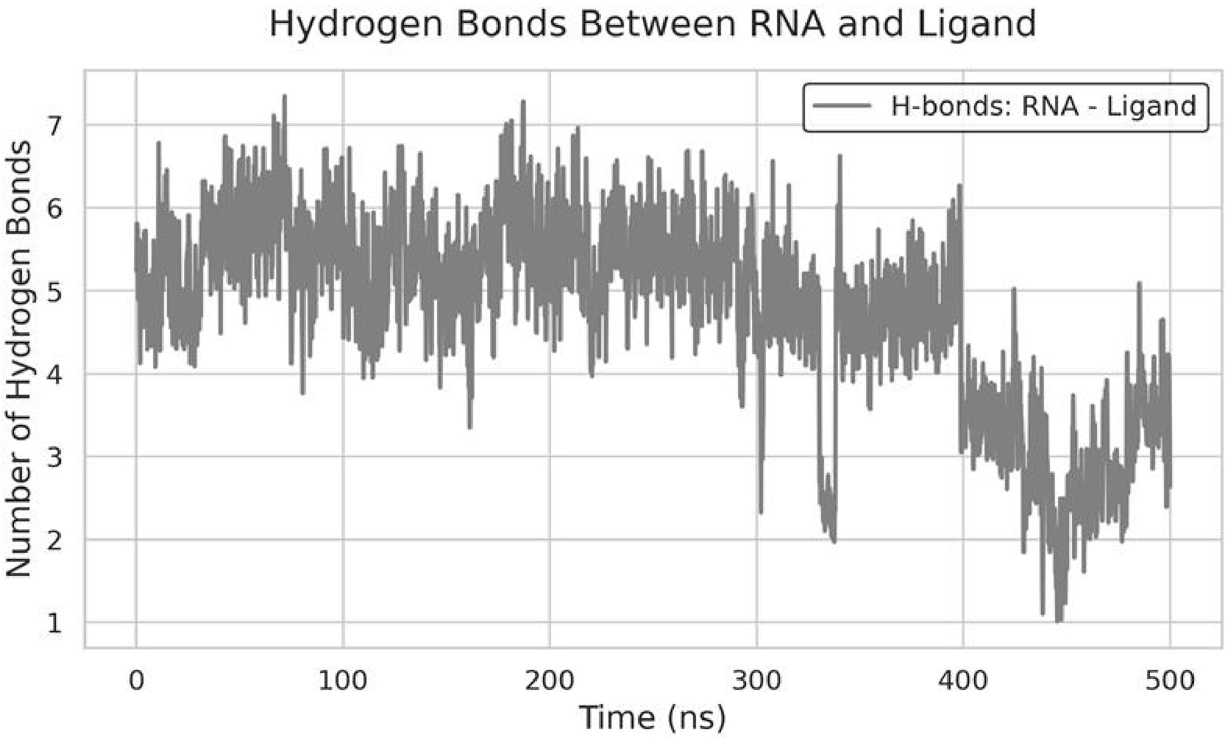

Figure S28: The number of hydrogen bonds for the N2 tautomer remained consistently around five throughout the 500 ns of plain MD simulations.

References

(1) Ghidini, A.; Serra, E.; Decherchi, S.; Cavalli, A. Bidirectional path-based non-equilibrium simulations for binding free energy. *Molecular Physics* **2024**, e2374465.

(2) Bertazzo, M.; Gobbo, D.; Decherchi, S.; Cavalli, A. Machine learning and enhanced sampling simulations for computing the potential of mean force and standard binding free energy. *Journal of chemical theory and computation* **2021**, *17*, 5287–5300.

(3) Wang, Y.; Parmar, S.; Schneekloth, J. S.; Tiwary, P. Interrogating RNA–small molecule interactions with structure probing and artificial intelligence-augmented molecular simulations. *ACS Central Science* **2022**, *8*, 741–748.

(4) Bochevarov, A. D.; Harder, E.; Hughes, T. F.; Greenwood, J. R.; Braden, D. A.; Philipp, D. M.; Rinaldo, D.; Halls, M. D.; Zhang, J.; Friesner, R. A. Jaguar: A high-performance quantum chemistry software program with strengths in life and materials sciences. *International Journal of Quantum Chemistry* **2013**, *113*, 2110–2142.
